# A Combination of Plant-Derived Extracts Modulates Nutrient-Responsive Metabolic Signalling in an In Vitro Gut–Liver–Adipose Model

**DOI:** 10.3390/nu18091393

**Published:** 2026-04-28

**Authors:** Francesca Uberti, Rebecca Galla, Simone Mulè, Francesca Parini, Claudio Molinari

**Affiliations:** 1 Department for Sustainable Development and Ecological Transition, University of Piemonte Orientale (UPO), 13100 Vercelli, Italy; simone.mule@uniupo.it (S.M.); claudio.molinari@uniupo.it (C.M.); 2 Noivita S.r.l.s., Spin Off, University of Piemonte Orientale (UPO), Strada Privata Curti n. 7, 28100 Novara, Italy; rebecca.galla@noivita.it (R.G.); francescaparini00@gmail.com (F.P.)

**Keywords:** GLP-1 signalling, plant-derived extracts, in vitro multi-organ model, lipid metabolism, metabolic modulation

## Abstract

**Background/Objectives**: Glucagon-like peptide-1 (GLP-1) is a nutritionally regulated incretin involved in the coordination of intestinal, hepatic, and adipose metabolic responses. Although plant-derived extracts are increasingly investigated for their metabolic effects, mechanistic evidence integrating multiple metabolic tissues remains limited. This study aimed to investigate the molecular effects of a combination of plant-derived extracts in an integrated in vitro gut–liver–adipose model. **Methods**: Differentiated Caco-2 monolayers were exposed to a standardised combination of plant-derived extracts obtained from *Gastrodia elata*, *Morus alba*, and *Paeonia lactiflora*. GLP-1 secretion and epithelial barrier integrity were assessed. Conditioned media from intestinal cells were applied to HepG2 hepatocytes, and downstream effects on lipid metabolism-related pathways were evaluated. Subsequently, conditioned media from hepatic cells were applied to differentiated 3T3-L1 adipocytes to assess lipid accumulation and metabolic signalling. **Results**: Exposure of intestinal cells to the extract combination significantly increased GLP-1 secretion without altering epithelial barrier integrity. Intestinal conditioned media were associated with reductions in intracellular triglyceride levels in hepatocytes and with modulation of markers linked to lipid handling, including resistin, FGF21, SREBP-1c, NRF2, Src, AMPK, SIRT1, and PGC1α, suggesting GLP-1-associated effects. In adipocytes, hepatic conditioned media decreased lipid accumulation and increased the levels of metabolic markers associated with adipocyte browning-related signalling, including UCP1, NOS, SIRT1, and STAT3. **Conclusions**: Within the limitations of this in vitro multi-organ model, these findings suggest that the tested combination of plant-derived extracts modulates cellular pathways related to GLP-1-associated metabolic signalling across intestinal, hepatic, and adipose systems. These results should be interpreted as mechanistic and hypothesis-generating, and further in vivo and clinical studies are required to confirm their physiological relevance.

## 1. Introduction

Metabolic homeostasis depends on coordinated interactions among the gut, liver, pancreas, and adipose tissue, regulating nutrient sensing, hormonal signalling, and energy partitioning. Disruption of this network under metabolic stress contributes to ectopic fat accumulation and dysfunction independently of overall body weight [1,2,3].

Visceral adipose tissue plays a central role in metabolic regulation due to its high lipolytic activity and direct drainage into the portal circulation. Excess lipid storage in visceral adipocytes contributes to hepatic lipid overload, insulin resistance, and low-grade inflammation, whereas favourable adaptations involve reduced lipid storage, enhanced fatty acid oxidation, and improved metabolic flexibility [4,5]. At the cellular level, these adaptations are associated with increased mitochondrial activity and induction of uncoupling protein 1 (UCP1), a marker of browning-related processes [6], representing early molecular events that often precede measurable changes in body weight [7].

Among endocrine pathways linking nutrient intake to systemic metabolism, the incretin Glucagon-like peptide-1 (GLP-1), secreted by intestinal L-cells in response to nutrients, represents a key mediator of gut–peripheral tissue communication. GLP-1-associated signalling has been linked to postprandial regulation of insulin secretion, appetite, lipid metabolism, and energy balance [8,9,10,11,12,13]. Its receptor (GLP-1R), expressed in pancreatic, hepatic, and adipose cells, may contribute to these coordinated responses.

In hepatic tissue, signalling pathways associated with GLP-1 have been reported to reduce de novo lipogenesis, limit intracellular triglyceride (TG) accumulation, and enhance metabolic adaptability through the coordinated activation of AMP-activated protein kinase (AMPK), sirtuin 1 (SIRT1), and peroxisome proliferator-activated receptor γ coactivator 1α (PGC1α) [14]. Conversely, decreased GLP-1 activity has been linked to the upregulation of lipogenic transcription factors, such as sterol regulatory element-binding protein 1c (SREBP-1c), ultimately leading to hepatic lipid deposition [15]. Given the liver’s crucial role in regulating lipid distribution to peripheral tissues, these hepatic changes are likely to have downstream effects on adipose tissue function.

Hepatic lipid handling is further linked to adipose tissue metabolism through the release of hepatokines that influence systemic energy balance [16,17]. Among these, resistin and fibroblast growth factor 21 (FGF21) exert opposing effects on lipid storage, inflammation, and metabolic flexibility [18]. Elevated resistin levels are associated with insulin resistance, inflammatory signalling, and enhanced lipid storage [19,20,21], whereas FGF21 promotes fatty acid oxidation, mitochondrial function, and adaptive responses to metabolic stress [22,23,24,25]. Notably, GLP-1-associated pathways have been reported to influence hepatic FGF21 production, suggesting that intestinal incretin signalling may indirectly modulate adipose tissue metabolism through liver–adipose communication [26,27,28,29]. The presence of GLP-1 receptors in adipocytes suggests a role for incretin-associated pathways in modulating lipogenesis, lipolysis, and inflammatory signalling [30]. Improved hepatic lipid processing can further limit lipid delivery to adipose depots, favouring a shift toward a more oxidative adipocyte phenotype, including increased mitochondrial activity and induction of browning-related molecular markers [31,32].

Despite the clinical efficacy of synthetic GLP-1R agonists, their use is frequently limited by gastrointestinal adverse effects and high costs [33]. Specific plant-derived compounds, including polyphenols, polysaccharides, and oligostilbenes, have been shown to stimulate GLP-1 secretion in enteroendocrine models [34], suggesting potential roles in metabolic regulation beyond glucose homeostasis. In this context, extracts from *Morus alba*, *Gastrodia elata*, and *Paeonia lactiflora* have individually demonstrated beneficial effects on glucose and lipid metabolism, intestinal function, oxidative stress, and inflammatory responses [35,36,37]. *Morus alba* has been reported to modulate GLP-1 levels, improve insulin sensitivity, and influence hepatic lipid metabolism [38]. *Gastrodia elata*, traditionally used for neurological indications, also impacts glucose and lipid regulatory pathways and may influence enteroendocrine signalling [39]. *Paeonia lactiflora*, via its active compound paeoniflorin, can stimulate GLP-1 secretion and activate AMPK-mediated energy pathways, while modulating adipocyte lipid accumulation and browning-related signalling [40]. Collectively, these botanicals were selected for their complementary effects on GLP-1-associated signalling and inter-tissue metabolic pathways. However, their biological effects on GLP-1–associated metabolic signalling across interconnected metabolic tissues remain poorly characterised.

Integrated in vitro multi-organ models, including gut–liver–adipose platforms, provide a valuable experimental framework to dissect early nutrient-driven and inter-tissue signalling mechanisms. While these systems cannot replicate whole-body energy balance or adipose tissue mass, they allow mechanistic investigation of how intestinal GLP–1–associated signalling may influence downstream hepatic lipid metabolism and adipocyte browning-related pathways, capturing molecular events that precede measurable changes in body weight or fat depots [41,42].

Accordingly, the aim of this study was to investigate the metabolic effects of a standardized combination of *Gastrodia elata*, *Morus alba*, and *Paeonia lactiflora* extracts using an integrated in vitro gut–liver–adipose model. Specifically, we examined whether nutritional modulation of endogenous GLP-1 secretion at the intestinal level could propagate downstream change in cellular metabolic pathways in hepatic and adipose cells, including pathways associated with lipid handling and adipocyte remodelling, and activation of browning-associated molecular pathways, without implying direct measurement of body weight or visceral fat mass. These outcomes are intended to provide mechanistic insight rather than evidence of physiological or clinical effects.

## 2. Materials and Methods

### 2.1. Phytochemical Characterisation of the Botanical Extracts

To ensure the quality, standardisation, and reproducibility of the botanical extracts used in this study, a targeted phytochemical characterisation was conducted using established and validated analytical methods. Since the composition of the extract plays a central role in determining biological activity, representative bioactive markers were selected for each botanical ingredient based on their documented relevance to metabolic health regulation.

The *Morus alba* leaf extract was prepared from the leaves of *Morus alba* L., focusing on plant parts rich in bioactive compounds such as flavonoids and 1-deoxynojirimycin (1-DNJ), which are implicated in carbohydrate metabolism and incretin-related pathways. 1-DNJ is a naturally occurring iminosugar with potent α-glucosidase inhibitory activity, widely studied for its hypoglycaemic and metabolic effects in both preclinical and clinical contexts [43,44]. 1-DNJ content was assessed using high-performance liquid chromatography (HPLC) after derivatisation, following a validated analytical protocol. Analytical approaches such as HPLC–MS/MS and HPLC-FLD (Agilent, Santa Clara, CA, USA) with pre-column derivatisation have been used to reliably quantify 1-DNJ in *Morus alba* leaves and related products, demonstrating sensitivity and specificity for this iminosugar [45].

The *Gastrodia elata* extract was analysed by determining its total polysaccharide content, as polysaccharides are a key bioactive component of this botanical and have been linked to metabolic and anti-inflammatory effects. Total polysaccharides were measured using the phenol–sulfuric acid colourimetric assay, a widely recognised method for assessing polysaccharides in plant-derived matrices [46].

Meanwhile, the *Paeonia lactiflora* extract was characterised by measuring its total polyphenol and flavonoid contents, which are commonly linked to antioxidant and metabolic regulatory roles. Total polyphenols were quantified using the Folin–Ciocalteu method [47], while total flavonoid content was determined with a colourimetric aluminium chloride-based assay [48]. The results obtained were within the range reported in the literature for high-quality botanical extracts, confirming the chemical consistency of the preparation.

Overall, this phytochemical profiling confirms that the botanical extracts used in the present study were properly standardised and contained representative levels of bioactive constituents relevant to metabolic regulation. Detailed analytical procedures, validation parameters, and comprehensive methodological information for phytochemical analyses are included in Appendix A.1, Appendix A.2, Appendix A.3 and Appendix A.4.

### 2.2. Agent Preparation

*Morus alba* L. (Leaf extract), *Gastrodia elata* Blume (Rhizoma extract), and *Paeonia lactiflora* (Root extract) were evaluated both as single treatments and as a combined formulation to investigate their impact on intestinal barrier function and metabolic signalling within the gut–liver–adipose axis. Concentration ranges were selected based on preliminary dose–response assessments and supported by the literature data describing the metabolic activity of the respective extracts [49].

**Figure A1:**
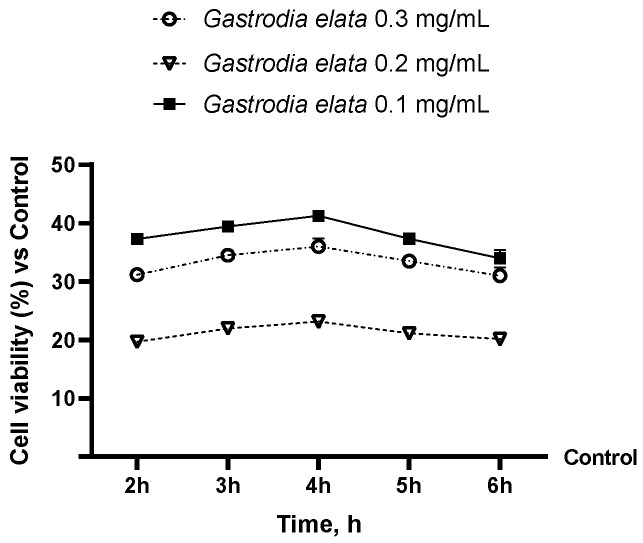
Cell viability assay of *Gastrodia elata* rhizome extract on intestinal cells (Caco-2). Data are expressed as mean ± SD (%) of 5 independent experiments, each performed in triplicate and normalised to the control (0%) line. *p* < 0.05 vs. control.

Specifically, *Morus alba* extract was administered at concentrations of 0.1–0.5 mg/mL, whereas *Paeonia lactiflora* extract was tested at 0.1–1 mg/mL. *Gastrodia elata* dry extract (10:1) was tested at a fixed concentration of 0.1 mg/mL, selected based on previous optimisation experiments and published dose–response evidence in Caco-2 cells [49] (Appendix A, Figure A1). Considering the observed efficacy and the absence of cytotoxic effects, optimal concentrations were selected and combined into a formulation comprising *Gastrodia elata* (0.1 mg/mL), *Morus alba* (0.2 mg/mL), and *Paeonia lactiflora* (0.1 mg/mL). All extracts were prepared as concentrated stock solutions by accurately weighing the dry materials and dissolving them in culture medium. The prepared stock solutions were subsequently diluted in phenol red-free Dulbecco’s Modified Eagle’s Medium (DMEM)supplemented with 0.5% fetal bovine serum (FBS), 2 mM L-glutamine, and 1% penicillin–streptomycin, which served as the working medium for all experimental assays (all from Merck Life Sciences, Rome, Italy). The botanical extracts utilised in this study were supplied by Noivita S.r.l. (Novara, Italy). For all experimental conditions, untreated cells cultured under identical conditions served as controls, representing the baseline physiological state.

### 2.3. Cell Cultures

Human intestinal epithelial Caco-2 cells and enteroendocrine NCI-H716 cells (American Type Culture Collection, ATCC^®^, Manassas, VA, USA) were used to create an in vitro intestinal model that mimics the epithelial–enteroendocrine interface, allowing simultaneous evaluation of epithelial transport and GLP-1 secretion. Caco-2 cells were cultured in Advanced Dulbecco’s Modified Eagle Medium (Adv-DMEM; GIBCO^®^, Thermo Fisher Scientific, Waltham, MA, USA) supplemented with 10% FBS, 2 mM L-glutamine, and 1% penicillin–streptomycin. The cultures were incubated at 37 °C under standard humidified conditions with 5% CO_2_ [44,50]. Cells between passages 26 and 32 were used to ensure stable differentiation and reproducible barrier properties [51]. Caco-2 cells were seeded onto polycarbonate Transwell^®^ inserts (6.5 mm diameter, 0.4 μm pore size; Corning^®^ Costar^®^, Merck Life Science, Rome, Italy) at a density of 1.5 × 10^4^ cells per insert and allowed to differentiate over 21 days, with medium changes every 48 h, to form a fully mature epithelial monolayer [52].

NCI-H716 cells were cultured in Roswell Park Memorial Institute (RPMI)-1640 medium (GIBCO^®^, Thermo Fisher Scientific) supplemented with 10% FBS, 2 mM L-glutamine, and 1% penicillin–streptomycin under standard incubation conditions. Cells between passages 20 and 35 were used to ensure phenotypic stability and consistent secretory activity, as previously described [53]. To establish the co-culture model, NCI-H716 cells were seeded at a density of 5 × 10^4^ cells per insert onto Transwell^®^ membranes coated with type I collagen, which contained fully differentiated Caco-2 monolayers. Collagen coating was utilised to promote adhesion and differentiation of NCI-H716 cells. Following seeding, NCI-H716 cells were allowed to adhere and differentiate for 48–72 h prior to experimental treatments, in accordance with previously validated protocols [54].

Human HepG2 hepatocellular carcinoma cells (ATCC^®^, Manassas, VA, USA) were used as an in vitro hepatic model to investigate glucose and lipid metabolic pathways. HepG2 cells were cultured in Adv-DMEM supplemented with 10% FBS, 2 mM L-glutamine, and 1% penicillin–streptomycin under standard conditions (37 °C, 5% CO_2_) and used for experiments upon reaching 90–95% confluence to ensure metabolic competence and reproducibility [55]. A total of 3.5 × 10^4^ cells were seeded into 24-well plates fitted with 6.5 mm Transwell^®^ inserts (Corning^®^ Costar^®^, Merck Life Science, Rome, Italy). The development of a mature monolayer was confirmed by TEER, which reached 486 Ω·cm^2^.

Murine 3T3-L1 preadipocytes (ATCC^®^, Manassas, VA, USA) were employed as an adipocyte model to evaluate lipid accumulation, adipogenic differentiation, and browning-related pathways. Cells were cultured in DMEM supplemented with 10% FBS, 2 mM L-glutamine, and 1% penicillin–streptomycin at 37 °C in 5% CO_2_. Cells were subcultured between passages 2 and 10 to preserve adipogenic potential [56,57]. Differentiation into mature adipocytes was induced using a standard hormonal cocktail, as previously described [58], and fully differentiated adipocytes were utilised for subsequent metabolic and browning analyses [59,60].

### 2.4. Experimental Protocol

**Figure 1:**
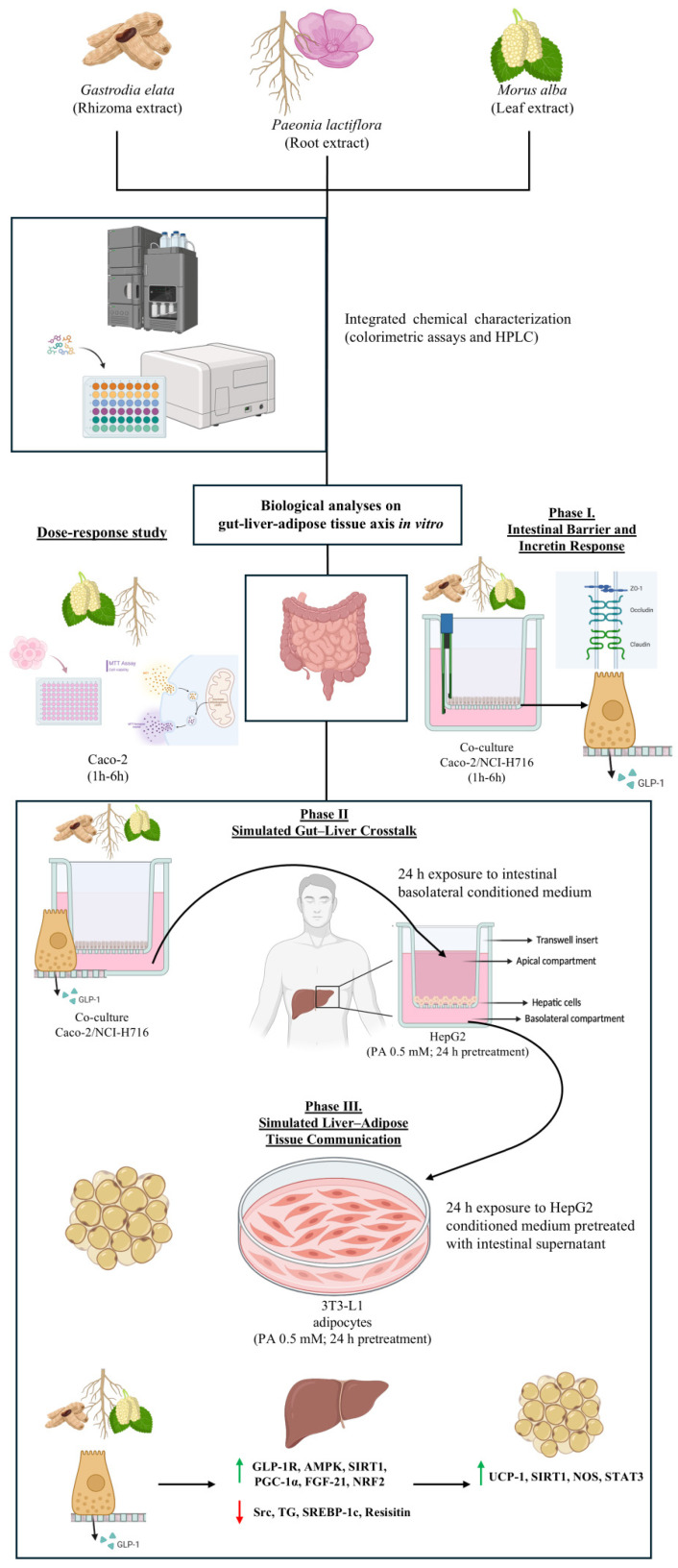
Illustrative scheme of the experimental protocol and the in vitro multi-organ pathway under investigation.

The experimental design was structured as a sequential in vitro model to replicate key aspects of the gut–liver–adipose axis and to examine the metabolic effects of the botanical combination along this integrated pathway. The protocol was organised into three interconnected phases, reflecting the putative propagation of intestinal signals towards hepatic and adipocyte metabolic responses (Figure 1). Before stimulation, all cell types were synchronised overnight in phenol red-free DMEM supplemented with L-glutamine, sodium pyruvate, and penicillin–streptomycin but without FBS. All experimental procedures were conducted under standard incubation conditions (37 °C, 5% CO_2_).

- Preliminary Phase. Chemical characterisation and Cytocompatibility-Dose Selection: an initial chemical characterisation of single extracts and intestinal cytocompatibility screening were performed. Cytocompatibility was analysed using a 3-(4,5-dimethylthiazol-2-yl)-2,5-diphenyltetrazolium bromide (MTT)-based assay (detailed in Appendix A.5) to identify non-cytotoxic concentrations of the individual extracts and their combination, ensuring cell viability across all experimental models prior to functional analyses [61]. This assessment was primarily conducted for *Morus alba* and *Paeonia lactiflora*, which required experimental concentration optimisation, while *Gastrodia elata* concentrations were selected based on previously published dose–response data and a prior study [49]. Based on these results, selected concentrations were used for all subsequent experiments.
- Phase I. Intestinal Barrier and Incretin Response: an in vitro intestinal barrier model based on a Transwell^®^ co-culture of Caco-2 and NCI-H716 cells was employed to assess epithelial integrity, permeability, and enteroendocrine function within the 1–6 h time window. Barrier maturation and integrity were monitored by transepithelial electrical resistance (TEER) measurements, while tight junction (TJ) functionality was evaluated through quantification of claudin-1, occludin, and Zonula Occludens-1 (ZO-1) proteins [51,52]. Intestinal permeability was assessed using a fluorescent tracer to evaluate transport kinetics across the epithelial layer [62]. Following stimulation with the botanical combination, basolateral supernatants were collected to quantify GLP-1 secretion and generate conditioned media for downstream experiments.
- Phase II. Simulated Gut–Liver Crosstalk: an in vitro intestine–liver axis was established to investigate hepatic metabolic responses to intestinal-derived signals. HepG2 cells were first exposed to palmitic acid (PA; Merck Life Sciences, Rome, Italy) 0.5 mM for 24 h to induce intracellular lipid accumulation and a steatosis-like condition. Subsequently, cells were treated for another 24 h with basolateral supernatants collected from the intestinal model. Hepatic responses were evaluated by analysing key signalling and metabolic markers involved in glucose and lipid regulation, including GLP-1R expression, intracellular TG accumulation, activation of AMPK-related pathways, and secretion of hepatokines associated with metabolic homeostasis, such as resistin and FGF-21.
- Phase III. Simulated Liver–Adipose Tissue Communication: the gut–liver–adipose axis was completed by exposing differentiated 3T3-L1 adipocytes, for 24 h, to conditioned media collected from HepG2 cells previously treated with intestinal supernatants. Prior to stimulation, as performed at the hepatic level, adipocytes were treated with PA to induce lipid accumulation and metabolic stress. Adipocyte responses were subsequently evaluated by assessing lipid accumulation, markers of adipogenic and lipogenic activity, and proteins associated with mitochondrial function and browning-related signalling, including UCP1 and related signalling pathways.

**Figure A2:**
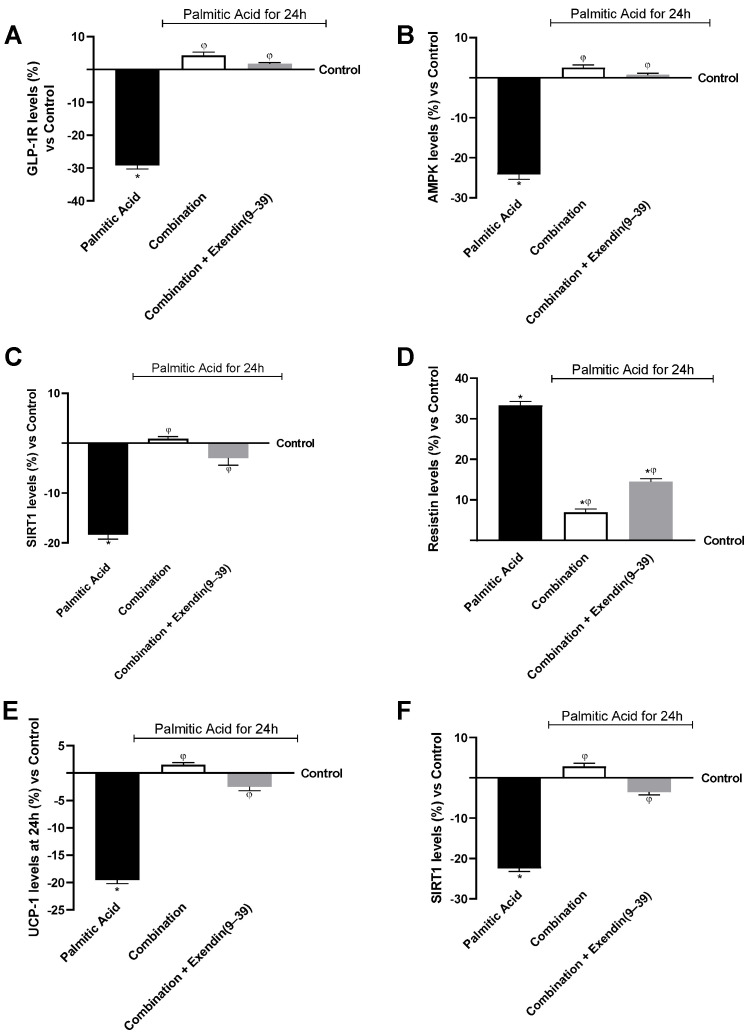
Mechanistic evaluation of combination treatment with the GLP-1R antagonist Exendin (9–39) on metabolic markers. Metabolic markers, including GLP-1R, AMPK, SIRT1, and resistin, were quantified in HepG2 cells, while SIRT1 and UCP-1 levels was evaluated in 3T3-L1 adipocytes. Specifically, in (**A**) GLP-1R levels; in (**B**) AMPK levels; in (**C**) SIRT1 levels (HepG2 cells); in (**D**) Resistin levels; in (**E**) UCP-1 levels; in (**F**) SIRT1 levels (3T3-L1 cells). The *Morus alba* extract used in this study was obtained from leaves, the *Paeonia lactiflora* extract from roots, and the *Gastrodia elata* extract from rhizomes. Data are expressed as mean ± SD (%) from 5 independent experiments, each performed in triplicate and normalised to the control (0%) line. * *p* < 0.05 vs. control; ^φ^ *p* < 0.05 vs. PA.

**Figure A3:**
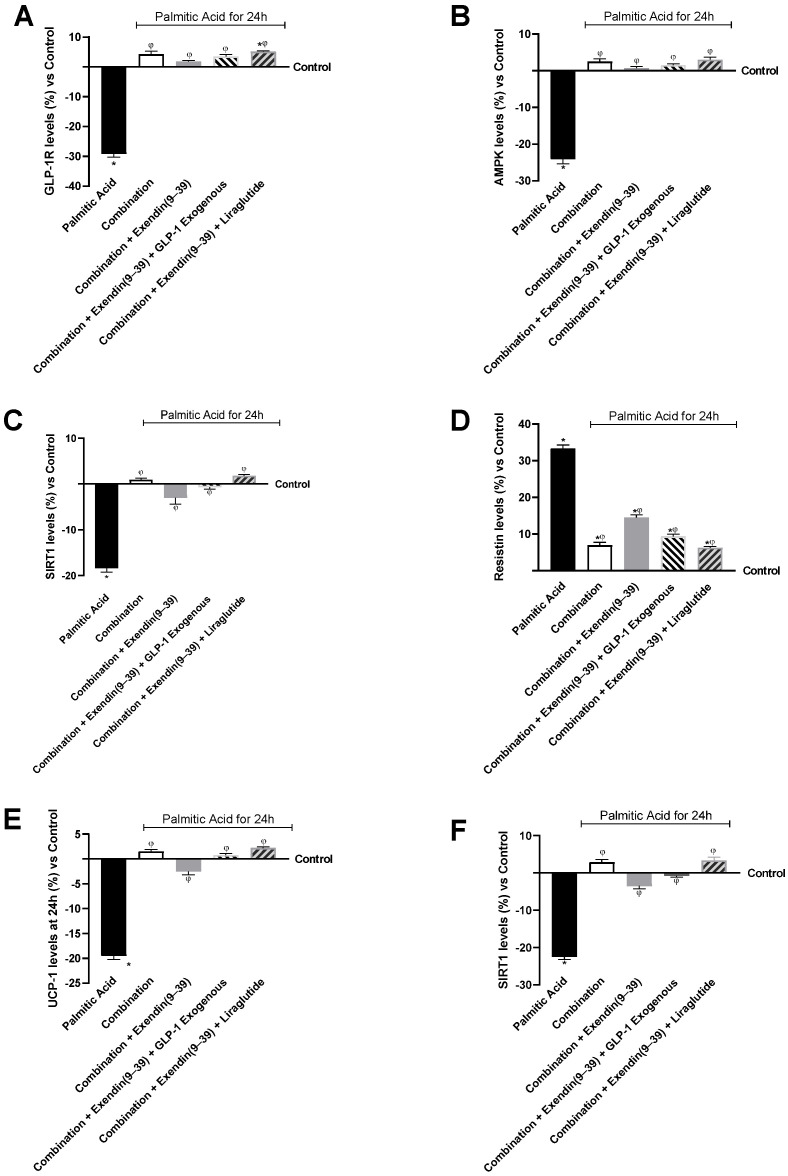
Validation of GLP-1R-mediated signalling through rescue experiments with exogenous GLP-1 and Liraglutide (both at 100 nM). The contribution of the GLP-1R pathway was confirmed by co-treating cells with the botanical combination and either exogenous GLP-1 or a specific GLP-1R agonist (Liraglutide) in the presence of the antagonist Exendin(9–39). Metabolic markers, including GLP-1R, AMPK, SIRT1, and resistin, were quantified in HepG2 cells, while SIRT1 and UCP-1 levels was evaluated in 3T3-L1 adipocytes. Specifically, in (**A**) GLP-1R levels; in (**B**) AMPK levels; in (**C**) SIRT1 levels (HepG2); and in (**D**) Resistin levels; in (**E**) UCP-1 levels and in (**F**) SIRT1 levels (3T3-L1). The *Morus alba* extract used in this study was obtained from leaves, the *Paeonia lactiflora* extract from roots, and the *Gastrodia elata* extract from rhizomes. Data are expressed as mean ± SD (%) of 5 independent experiments, each performed in triplicate and normalised to the control (0%) line. * *p* < 0.05 vs. control; ^φ^ *p* < 0.05 vs. PA.

To investigate the contribution of GLP-1R signalling to the downstream effects observed in our multi-organ model, additional experiments were performed using the GLP-1R antagonist Exendin(9–39) (Merck Life Science, Rome, Italy). Exendin(9–39) is a well-characterised competitive antagonist of the GLP-1 receptor shown to effectively inhibit GLP-1-mediated signalling in vitro [63]. HepG2 and 3T3-L1 cells were pretreated with Exendin(9–39) at a final concentration of 500 nM for 45 min prior to exposure to conditioned media with the botanical combination. Furthermore, to evaluate receptor mechanistic specificity through a “rescue” approach, additional sets of cells were co-treated with the botanical combination and either exogenous human GLP-1 (100 nM; Merck Life Science, Rome, Italy) or a specific GLP-1R agonist (Liraglutide, 100 nM; Merck Life Science, Rome, Italy) in the presence of the antagonist [64,65]. Following antagonist pretreatment, cells were processed as described above for measurement of intracellular triglyceride content and analysis of target markers, including GLP-1R, AMPK, SIRT1, resistin, and UCP1 levels through an ELISA kit. Data derived from these antagonist experiments and rescue are presented in Appendix A (Figure A2 and Figure A3).

### 2.5. In Vitro Intestinal Barrier Model

To evaluate intestinal absorption, permeability, and potential bioavailability of the tested extracts and their combinations, an in vitro barrier model was created using a Transwell^®^ system with Caco-2 and NCI-H716 cells. This method aligns with widely accepted approaches for intestinal modelling and corresponds with frameworks referenced by regulatory agencies such as the Food and Drug Administration (FDA) and the European Medicines Agency (EMA), as well as with established protocols described in the literature [52,66,67].

Caco-2 cells were cultured and differentiated on Transwell^®^ inserts as previously described, with medium renewed in both the apical and basolateral compartments every 48 h until full epithelial maturation was achieved [52].

The formation of a functional epithelial barrier was evaluated by measuring transepithelial electrical resistance (TEER) using an EVOM 3 volt-ohmmeter equipped with STX 2 electrodes (World Precision Instruments, Sarasota, FL, USA). Only cell monolayers with TEER values of at least 400 Ω·cm^2^ on day 21 were selected for further permeability studies [62]. To simulate physiological intestinal conditions during stimulation, the apical compartment was adjusted to pH 6.5, while the basolateral side was maintained at pH 7.4, reflecting luminal and systemic environments, respectively [51]. Cells were exposed to the selected extracts and their combination for 1–6 h. Intestinal permeability was assessed using fluorescein (0.04%; Santa Cruz Biotechnology, Santa Cruz, CA, USA) as a paracellular tracer. After a 40-min incubation at 37 ° C, fluorescein transport to the basolateral compartment was quantified using a fluorescence microplate reader (Infinite 200 Pro MPlex, Tecan, Männedorf, Switzerland) at excitation/emission wavelengths of 490/514 nm, respectively [62].

The permeation rate (J) was calculated based on Michaelis–Menten kinetics as:

J = Jmax × [C]/(Kt + [C])(1)

where:

- Jmax denotes the maximum permeation rate;
- [C] the initial fluorescein concentration;
- Kt the Michaelis–Menten constant.

Negative controls, consisting of Transwell^®^ inserts without cells, were included to exclude nonspecific membrane permeation.

At the end of the 6 h incubation, basolateral supernatants were collected to quantify GLP-1 secretion and to treat hepatic and adipocyte cell models.

### 2.6. TJ Protein Analysis

The integrity and function of the intestinal epithelial barrier after treatment were confirmed by measuring the levels of key TJ proteins in Caco-2/NCI-H716 co-cultures. Specifically, Claudin-1, Occludin, and ZO-1 levels were quantified as established markers of epithelial junctional organisation and paracellular permeability [51]. Following experimental stimulation, cell lysates were collected and analysed using commercially available ELISA kits for Claudin-1 (Cusabio Technology LLC, Houston, TX, USA), Occludin (MyBioSource, San Diego, CA, USA), and ZO-1 (MyBioSource, San Diego, CA, USA), following the manufacturers’ instructions [51]. Absorbance was measured at 450 nm using a microplate reader (Infinite 200 Pro MPlex, Tecan, Männedorf, Switzerland). Protein concentrations were determined based on standard calibration curves (0–1000 pg/mL for Claudin-1 and ZO-1; 0–1500 pg/mL for Occludin), and results were expressed as percentage changes relative to untreated controls. Data are presented as mean ± SD (%) from five independent experiments, each conducted in triplicate, and relative to control values (0% reference line).

### 2.7. ELISA Assay for GLP-1

Glucagon-like peptide-1 (GLP-1) secretion was measured in basolateral supernatants collected from the Caco-2/NCI-H716 Transwell^®^ co-culture system after 6 h of stimulation. GLP-1 levels were analysed using a commercial Human GLP-1 ELISA kit (Thermo Fisher Scientific, Waltham, MA, USA), following the manufacturer’s instructions and previously validated methods [53,54]. Optical density was read at 450 nm with a microplate reader (Infinite 200 Pro MPlex, Tecan, Männedorf, Switzerland). Concentrations were calculated from standard calibration curves and expressed as percentage changes relative to untreated controls. Results are presented as mean ± SD (%) from five independent experiments performed in triplicate.

### 2.8. ELISA Assay for Src Phosphorylation

The activation state of the proto-oncogene tyrosine-protein kinase Src (Src) was assessed in HepG2 cells after exposure to conditioned media from the intestine, to examine early intracellular signalling events associated with GLP-1 pathways. Src phosphorylation was measured using cell-based ELISA kits specific for total Src and Src phosphorylated at tyrosine 529 (pSrc Tyr^529^) (MyBioSource, San Diego, CA, USA), following the manufacturer’s instructions and established literature protocols. HepG2 cells were seeded in 96-well plates at suitable densities and exposed to the indicated treatments. At the end of the incubation period, cells were fixed using the supplied fixation solution to preserve phosphorylation states. A primary antibody specific for Src phosphorylated at Tyr529 was then applied, followed by incubation with an HRP-conjugated secondary antibody. Signal detection was performed through enzymatic colour development with the provided substrate, and absorbance was measured at 450 nm using a microplate reader (Infinite 200 Pro MPlex, Tecan, Männedorf, Switzerland). Simultaneously, total Src protein levels were determined using a dedicated ELISA kit capable of detecting all Src isoforms regardless of their phosphorylation status. For each experimental condition, the ratio of phosphorylated Src to total Src (pSrc/Src) was calculated as an index of Src activation. Data are presented as mean ± SD (%) from five independent experiments, each conducted in triplicate, and relative to control values (0% reference line).

### 2.9. ELISA Assay for GLP-1R

GLP-1R expression was measured in HepG2 cell lysates to assess hepatic sensitivity to intestinally derived GLP-1 signalling after exposure to conditioned media. GLP-1 receptor (GLP-1R) levels were quantified using a commercially available Human GLP-1R ELISA kit (FineTest, Wuhan, China), following the manufacturer’s protocol. Optical density was measured at 450 nm with a microplate reader (Infinite 200 Pro MPs, Tecan, Männedorf, Switzerland). Protein levels were derived from the corresponding standard calibration curve and are presented as mean ± SD (%) relative to untreated control cells. Data are presented as mean ± SD (%) from five independent experiments, each conducted in triplicate, and relative to control values (0% reference line).

### 2.10. TG Colourimetric Assay

Intracellular TG accumulation was measured in HepG2 cells to assess hepatic lipid processing after exposure to intestinal-derived conditioned media. TG levels were determined using a TG Colourimetric Assay Kit (Invitrogen™, Thermo Fisher Scientific), following the manufacturer’s instructions. At the end of the stimulation period, HepG2 cells were lysed, and 2.5 μL of each sample was transferred to a 96-well plate. After incubation at room temperature for approximately 30 min, absorbance was measured at 510 nm using a microplate reader (Infinite 200 Pro MPlex, Tecan, Männedorf, Switzerland). TG levels were calculated using a standard calibration curve spanning 0.14 to 10 mmol/L. Data are presented as mean ± SD (%) from five independent experiments, each conducted in triplicate, and relative to control values (0% reference line).

### 2.11. ELISA Assay for SREBP-1c

SREBP-1c levels were measured in 3T3-L1 adipocyte lysates to evaluate the regulation of lipogenic transcription pathways after treatment with conditioned media from the intestinal-hepatic axis. SREBP-1c expression was assessed using a commercially available ELISA kit (LSBio, Seattle, DC, USA), according to the manufacturer’s instructions. The optical signal was read at 450 nm with a microplate reader (Infinite 200 Pro MPlex, Tecan, Männedorf, Switzerland). SREBP-1c levels were determined using a standard calibration curve ranging from 0.312 to 20 ng/mL. Data are presented as mean ± SD (%) from five independent experiments, each conducted in triplicate, and relative to control values (0% reference line).

### 2.12. ELISA Assay for AMPK Phosphorylation

AMPK activation was evaluated in HepG2 cell lysates to investigate downstream metabolic signalling pathways associated with GLP-1–mediated effects following exposure to intestinal-derived conditioned media. Levels of phosphorylated AMPK (Thr172) were measured using a Human AMPK α1/2 (Phospho) [pT172] ELISA Kit (Thermo Fisher Scientific, Milan, Italy), according to the manufacturer’s instructions and previously established protocols [68]. Absorbance at 450 nm was recorded with a microplate reader (Infinite 200 Pro MPlex, Tecan, Männedorf, Switzerland). Phosphorylated AMPK levels were calculated using the standard calibration curve supplied with the assay kit and are expressed as mean ± SD (%) relative to untreated control cells. Data are presented as mean ± SD (%) from five independent experiments, each conducted in triplicate, and relative to control values (0% reference line).

### 2.13. ELISA Assay for PGC1α

PGC1α levels were measured in HepG2 cell lysates to evaluate mitochondrial and oxidative metabolic responses downstream of AMPK activation after exposure to conditioned media derived from the intestine. PGC1α expression was quantified using a commercially available ELISA kit (Antibodies, Stockholm, Sweden), according to the manufacturer’s instructions. The optical signal at 450 nm was recorded with a microplate reader (Infinite 200 Pro MPlex, Tecan, Männedorf, Switzerland). PGC1α levels were determined from a calibration curve ranging from 0 to 10 ng/mL and are expressed as mean ± SD (%) relative to untreated control cells. Data are presented as mean ± SD (%) from five independent experiments, each conducted in triplicate, and relative to control values (0% reference line).

### 2.14. ELISA Assay for Resistin

Resistin levels were measured in HepG2 cell culture supernatants to assess the modulation of hepatokine release related to lipid metabolism and inflammatory signalling after exposure to intestinal-derived conditioned media. Resistin concentrations were determined using a commercially available Human Resistin ELISA Kit (Thermo Fisher Scientific, Milan, Italy), following the manufacturer’s instructions. The optical density at 450 nm was measured with a microplate reader (Infinite 200 Pro MPlex, Tecan, Männedorf, Switzerland). Resistin levels were quantified based on a standard calibration curve ranging from 31 to 2000 pg/mL. Data are presented as mean ± SD (%) from five independent experiments, each conducted in triplicate, and relative to control values (0% reference line).

### 2.15. ELISA Assay for FGF-21

Fibroblast growth factor 21 (FGF-21) levels were measured in HepG2 cell culture supernatants to evaluate hepatokine responses related to metabolic flexibility and lipid oxidation after exposure to intestinal-derived conditioned media. FGF-21 concentrations were determined using a Human FGF-21 DuoSet ELISA Kit (R&D Systems, Minneapolis, MN, USA), following the manufacturer’s instructions and established protocols [69]. Measurements at 450 nm were obtained using a microplate reader (Infinite 200 Pro MPlex, Tecan, Männedorf, Switzerland). FGF-21 levels were estimated from a standard calibration curve covering a range of 31.2–2000 pg/mL. Data are presented as mean ± SD (%) from five independent experiments, each conducted in triplicate, and relative to control values (0% reference line).

### 2.16. ELISA Assay for NRF2

Nuclear factor erythroid 2–related factor 2 (NRF2) levels were measured in HepG2 cell lysates to assess the activation of antioxidant and cytoprotective pathways involved in metabolic adaptation following exposure to intestinal-derived conditioned media. NRF2 expression was quantified using a commercially available NRF2 Quantification ELISA Kit (MyBioSource, San Diego, CA, USA) according to the manufacturer’s instructions and previously described methods [70]. The signal at 450 nm was measured using a microplate reader (Infinite 200 Pro MPlex, Tecan, Männedorf, Switzerland). NRF2 levels were determined from a calibration curve spanning 0–25 ng/mL. Data are presented as mean ± SD (%) from five independent experiments, each conducted in triplicate, and relative to control values (0% reference line).

### 2.17. Western Blot Analysis for UCP1

UCP1 expression was assessed in differentiated 3T3-L1 adipocytes to evaluate the activation of thermogenic and browning-related pathways following treatment with conditioned media derived from the intestinal-hepatic axis. UCP1 protein levels were analysed using Western blotting according to standard procedures. Briefly, 3T3-L1 adipocytes were lysed on ice with Complete Tablet lysis buffer (Roche, Basel, Switzerland) supplemented with 2 mM sodium orthovanadate (Na_3_VO_4_), 1 mM phenylmethylsulfonyl fluoride (PMSF; Merck Life Science, Rome, Italy), phosphatase inhibitor cocktail (1:50), and protease inhibitor cocktail (1:200; Merck Life Science, Rome, Italy). Total protein content was initially measured, and equal amounts of protein (35 μg per sample) were separated by sodium dodecyl sulphate–polyacrylamide gel electrophoresis (SDS–PAGE) using 10% polyacrylamide gels. After electrophoretic separation, proteins were transferred onto polyvinylidene difluoride (PVDF) membranes (GE Healthcare Europe GmbH). Membranes were incubated overnight at 4 °C with a primary antibody against UCP1 (1:500; Santa Cruz Biotechnology, Santa Cruz, CA, USA), while β-actin (1:4000, Merck Life Science, Rome, Italy) was used as a loading control to normalise protein expression. Immunoreactive bands were detected using appropriate secondary antibodies and visualisation reagents. Band intensity was quantified by densitometric analysis (Image Lab™ Software version 5.2.1; Bio-Rad, Hercules, CA, USA). UCP1 data expression are presented as mean ± SD (%) from three independent experiments, each conducted in triplicate, and relative to control values (0% reference line).

### 2.18. ELISA Assay for SIRT1

SIRT1 levels were quantified in HepG2 and differentiated 3T3-L1 cell lysates to assess the involvement of NAD^+^-dependent deacetylase signalling in metabolic regulation downstream of intestinal–hepatic communication. SIRT1 expression was measured using a commercially available Human SIRT1 ELISA Kit (Thermo Fisher Scientific™, Waltham, MA, USA) according to the manufacturer’s instructions and established protocols [71]. Absorbance readings at 450 nm were obtained using a microplate reader (Infinite 200 Pro MPlex, Tecan, Männedorf, Switzerland). SIRT1 levels were quantified based on a calibration curve ranging from 1.23 to 300 ng/mL. Data are presented as mean ± SD (%) from five independent experiments, each conducted in triplicate, and relative to control values (0% reference line).

### 2.19. ELISA Assay for eNOS/NOS3

Endothelial nitric oxide synthase (eNOS/NOS3) levels were measured in differentiated 3T3-L1 adipocyte lysates to evaluate nitric oxide-related signalling involved in mitochondrial function, metabolic adaptation, and adipose tissue browning after treatment with conditioned media from the intestinal–hepatic axis. eNOS expression was determined using a commercially available Human eNOS/NOS3 ELISA Kit (Invitrogen™, Thermo Fisher Scientific), following the manufacturer’s instructions. Measurements at 450 nm were obtained using a microplate reader (Infinite 200 Pro MPlex, Tecan, Männedorf, Switzerland). eNOS levels were determined from a calibration curve covering the range of 0.4–100 ng/mL. Data are presented as mean ± SD (%) from five independent experiments, each conducted in triplicate, and relative to control values (0% reference line).

### 2.20. ELISA Assay for STAT3

Signal transducer and activator of transcription 3 (STAT3) activation was assessed in differentiated 3T3-L1 adipocyte lysates to evaluate downstream signalling events related to adipose tissue remodelling and thermogenic responses following treatment with conditioned media from the intestinal-hepatic axis. Phosphorylated STAT3 at tyrosine 705 (pSTAT3 Tyr^705^) was measured using a commercially available STAT3 [pY705] ELISA Kit (Invitrogen™, Thermo Fisher Scientific), following the manufacturer’s instructions. Signal intensity at 450 nm was recorded using a microplate reader (Infinite 200 Pro MPlex, Tecan, Männedorf, Switzerland). Levels of phosphorylated STAT3 were quantified using the assay kit’s calibration curve. Data are presented as mean ± SD (%) from five independent experiments, each conducted in triplicate, and relative to control values (0% reference line).

### 2.21. Mouse Mitochondrial Brown Fat Uncoupling Protein 1 (UCP1) ELISA Kit

UCP1 levels in 3T3-L1 adipocyte lysates were quantified using a Cusabio ELISA kit (CUSABIO Innovation Center, Houston, TX, USA), following the manufacturer’s protocol. The enzymatic reaction was terminated by adding 50 μL of stop solution, and the resulting signal was measured at 450 nm using a Tecan Infinite 200Pro MPlex microplate reader (Männedorf, Switzerland). UCP1 concentrations were determined from a standard calibration curve (0–300 pg/mL). Data are presented as mean ± SD (%) from five independent experiments, each conducted in triplicate, and relative to control values (0% reference line).

### 2.22. Statistical Analysis

All data are reported as mean ± SD (%) and were normalised to untreated control values (0% reference line), except for TEER values and GLP-1 levels secretion.

Results are derived from at least five independent biological experiments, each performed with three technical replicates unless otherwise specified. For Western blot analyses, densitometric values are expressed as mean ± SD from three independent biological replicates, each conducted in triplicate, and representative images of the blots are provided. Biological replicates correspond to independent experimental runs conducted on different days and with different sample preparations, thereby capturing true biological variability.

Data distribution was assessed for normality using the Shapiro–Wilk test, while homogeneity of variance was evaluated using Levene’s test. Statistical comparisons among multiple groups were performed using one-way ANOVA followed by Bonferroni’s post hoc test. When the assumptions for parametric testing were not met, nonparametric analyses (Kruskal–Wallis test with Dunn’s post hoc test) were used to confirm the consistency of the observed trends.

Given the exploratory and mechanistic nature of the study, statistical interpretation was focused on identifying consistent biological patterns rather than drawing definitive conclusions on clinical relevance. The selected sample size (*n* = 5 biological replicates) was based on preliminary estimates of variability and is consistent with commonly accepted practices for in vitro mechanistic studies [72].

All statistical analyses were performed using GraphPad Prism (version 10.6.1; GraphPad Software, La Jolla, CA, USA). A *p*-value < 0.05 was considered statistically significant. The synergistic interactions were assessed post hoc using the Bliss independence model on specific biological markers. The expected additive response was derived from the effects of each single agent and subsequently compared with the experimentally observed combined outcome.

## 3. Results

### 3.1. Phytochemical Characterisation of the Plant-Derived Extracts

Prior to biological evaluation, the plant-derived extracts were chemically characterised to confirm their phytochemical profiles and degree of standardisation (Table 1). This preliminary assessment aimed to ensure consistency and quality of the materials used in subsequent in vitro experiments. High-performance liquid chromatography (HPLC) analysis verified that the *Morus alba* leaf extract contained a high level of 1-DNJ (5.03 ± 0.26 mg/g) and measurable levels of polysaccharides (12.00 ± 0.94 mg/g). In the absence of quantitative specifications in the analytical certificates for *Paeonia lactiflora* root and *Gastrodia elata* rhizome extracts, the contents of major phytochemical classes, including total polyphenols, flavonoids, and polysaccharides, as reported in Table 1, *Paeonia lactiflora* root extract contained total polyphenols (5.00 ± 0.43 mg/g) and flavonoids (0.70 ± 0.17 mg/g), determined using the Folin–Ciocalteu and AlCl_3_ methods, respectively. *Gastrodia elata* rhizome extract, on the other hand, contained total polysaccharides (10.20 ± 0.82 mg/g), measured by the phenol–sulfuric acid method, and total polyphenols (2.00 ± 0.34 mg/g), assessed with the Folin–Ciocalteu method. The phytochemical profiles obtained revealed distinct compositional features among the three extracts, supporting their complementary biological potential. Overall, this characterisation confirmed a sufficient level of standardisation and provided a rational basis for interpreting the downstream biological effects observed in the integrated in vitro model.

### 3.2. Selection of Non-Cytotoxic and Biologically Active Concentrations in Intestinal Cells

Before assessing intestinal barrier function and metabolic signalling, a dose–response study was performed in differentiated Caco-2 cells to identify non-cytotoxic and biologically active concentrations of the individual extracts and their combination. Cell viability was assessed over 1 to 6 h to exclude acute cytotoxic effects and to determine appropriate exposure conditions for subsequent functional assays.

**Figure 2:**
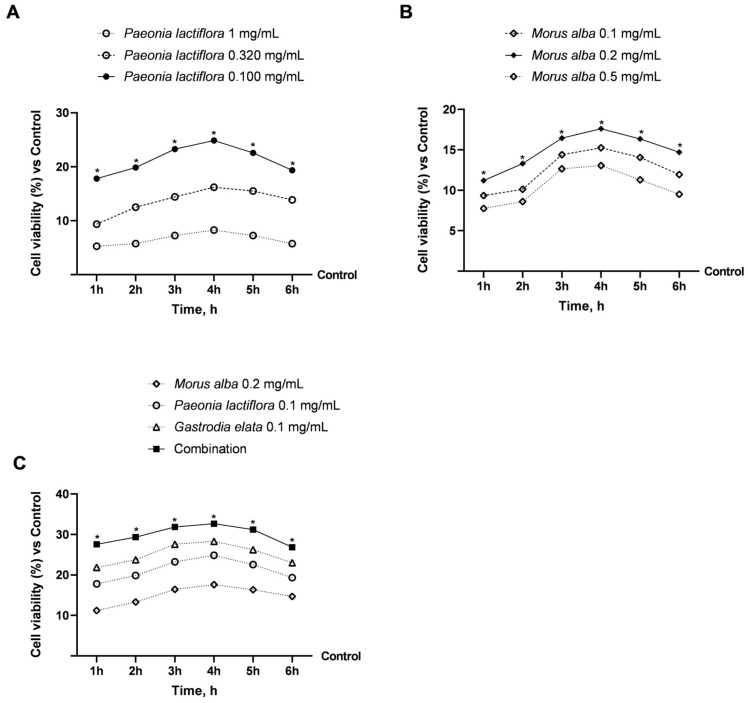
Effects of individual extracts on cell viability in Caco-2 cells under dose–response conditions, and effects of their combination under time-dependent conditions (1–6 h). In (**A**), dose–response effects of *Paeonia lactiflora* root extract; in (**B**), dose–response effects of *Morus alba* leaf extract; and in (**C**), comparative effects of the combination (*Gastrodia elata* + *Morus alba* + *Paeonia lactiflora*) versus individual components. *Gastrodia elata* concentration (0.1 mg/mL) were based on published dose–response data as described in Section 2.2 and reported in Appendix A. Data are expressed as mean ± SD (%) from 5 independent experiments, each performed in triplicate and normalized to the control (0%). * *p* < 0.05 vs. control.

As shown in Figure 2A,B, treatment with *Paeonia lactiflora* and *Morus alba* extracts did not induce cytotoxicity at any of the tested concentrations. On the contrary, both extracts significantly increased mitochondrial metabolic activity compared with untreated controls (*p* < 0.05).

For *Paeonia lactiflora*, the lowest concentration tested (0.1 mg/mL) was associated with the greatest increase, resulting in an approximately 25% increase in cell viability at 4 h, which was significantly greater than that observed at higher concentrations (*p* < 0.05). Similarly, *Morus alba* displayed maximal efficacy at an intermediate concentration of 0.2 mg/mL, leading to an approximately 18% increase in cell viability at 4 h (*p* < 0.05), whereas both lower and higher concentrations were less effective.

The combined formulation containing *Gastrodia elata* 0.1 mg/mL, *Morus alba* 0.2 mg/mL, and *Paeonia lactiflora* 0.1 mg/mL was subsequently evaluated and compared with the individual extracts (Figure 2C). The combination was associated with the greatest increase in cell viability, reaching a peak increase of approximately 33% at 4 h (*p* < 0.05), while exhibiting a kinetic profile comparable to that of the individual components. Importantly, no reduction in cell viability was observed at any time point, confirming the absence of cytotoxic effects.

Based on these results, the concentrations that maximised the observed increase in mitochondrial activity while preserving cell viability were selected for subsequent experiments: *Paeonia lactiflora* at 0.1 mg/mL, *Morus alba* at 0.2 mg/mL, and the combined extract formulation (hereinafter indicated as Combination).

### 3.3. Regulation of GLP-1 Secretion and Intestinal Barrier Integrity: The Primary Trigger of the Metabolic Axis

To establish the role of the gut as the initiating node of the metabolic axis, it was employed an in vitro co-culture model (Caco-2 and NCI-H716) to evaluate both the individual plant extracts and their combination. We focused on the gut’s sensing capacity by assessing GLP-1 secretion, alongside its structural response, including intestinal integrity (TEER), permeability, and the expression of TJ proteins.

**Figure 3:**
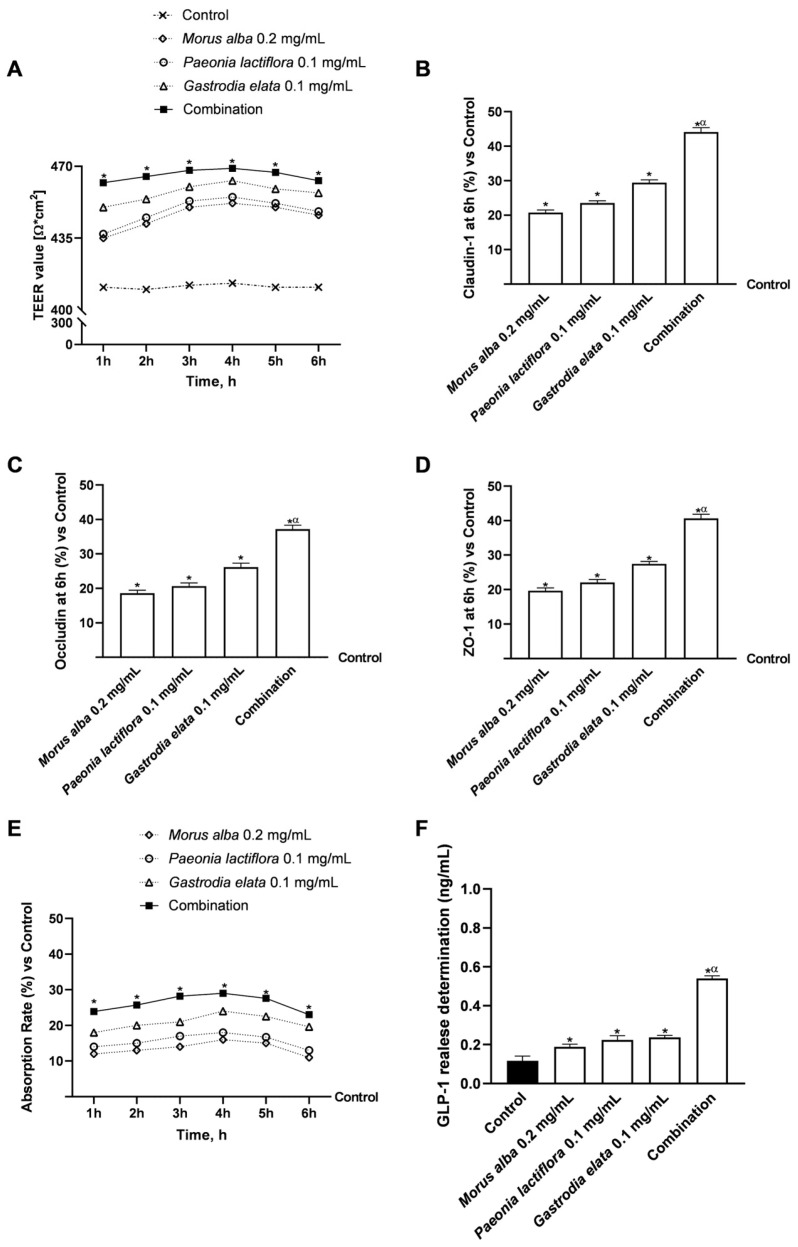
Evaluation of intestinal permeability in Caco-2 cells. (**A**) TEER values measured using EVOM3 voltohmmeter over time (1–6 h). (**B**–**D**) TJ protein levels (Claudin-1, Occludin, and ZO-1, respectively) were quantified by ELISA after 6 h of treatment. (**E**) Absorption rate over time (1–6 h), calculated according to the equation J = Jmax [C]/(Kt + [C]) using fluorescent probe. (**F**) GLP-1 secretion was measured after 6 h by ELISA kit. The botanical extracts used in this study were derived from *Morus alba* leaves, *Paeonia lactiflora* roots, and *Gastrodia elata* rhizomes. Data in panel (**A**) and (**F**) are presented as mean ± SD from five independent experiments each performed in triplicate. Data in panels (**B**–**E**) are expressed as mean ± SD from 5 independent experiments, each performed in triplicate and normalized to the control (0%).* *p* < 0.05 vs. control; ^α^ *p* < 0.05 vs. single components.

As shown in Figure 3A, treatment with all extracts preserved epithelial integrity throughout the experimental period, as indicated by stable or increased TEER values compared with untreated controls (*p* < 0.05). Among the individual components, *Gastrodia elata* 0.1 mg/mL exerted the most pronounced effect on maintaining barrier resistance. Notably, the combination produced the highest TEER values, reaching approximately 468 Ω·cm^2^ at 4 h, which were significantly greater than those of both the control and single-extract treatments (*p* < 0.05).

These observations were further supported by the analysis of TJ proteins. As shown in Figure 3B–D, the combined formulation significantly increased the expression of Claudin-1, Occludin, and ZO-1 compared with both control and individual extract treatments (*p* < 0.05). While *Gastrodia elata* alone displayed the strongest effect among the single agents, the combined treatment induced a markedly greater upregulation of all TJ markers, indicating greater-than-additive enhancement of epithelial cohesion.

Consistent with the improved barrier function, intestinal absorption analysis (Figure 3E) indicated that the combined formulation exhibited the highest transport efficiency across the epithelial monolayer. At 4 h, the combination achieved an absorption rate of approximately 29%, which was significantly higher than that observed for the individual extracts (*p* < 0.05).

Finally, the metabolic relevance of intestinal exposure was assessed by measuring GLP-1 secretion (Figure 3F). All treatments significantly stimulated GLP-1 release compared with control conditions (*p* < 0.05); however, the combined formulation elicited a markedly greater response, resulting in approximately 0.6 ng/mL of GLP-1 secretion. This level was significantly higher than those induced by *Gastrodia elata* (~0.22 ng/mL), *Paeonia lactiflora* (~0.21 ng/mL), or *Morus alba* (~0.18 ng/mL) alone (*p* < 0.05), without evidence of impaired barrier integrity.

Collectively, these findings indicate that the combined plant-derived formulation modulates intestinal barrier-related parameters and GLP-1 secretion in this in vitro model, supporting its potential role in modulating gut-derived metabolic signalling at the cellular level.

### 3.4. Integration of Intestinal Signals in Hepatic Metabolism: From GLP-1 Sensitivity to Lipid and Hepatokine Regulation

To evaluate whether the effects observed in the intestinal model could influence hepatic metabolic pathways, HepG2 cells were used to model liver responses. Lipotoxic stress was induced by exposing cells to PA (0.5 mM for 24 h) to model the metabolic alterations typical of excess lipid availability. To mimic the potential inter-organ communication, PA-stressed HepG2 cells were then incubated with the basolateral conditioned media collected from the intestinal Transwell^®^ system. This experimental design allowed us to investigate how the metabolites and signalling molecules, presumably generated during the epithelial transit of the extracts, might act as putative messengers to modulate hepatic lipid regulation in an integrated in vitro environment.

**Figure 4:**
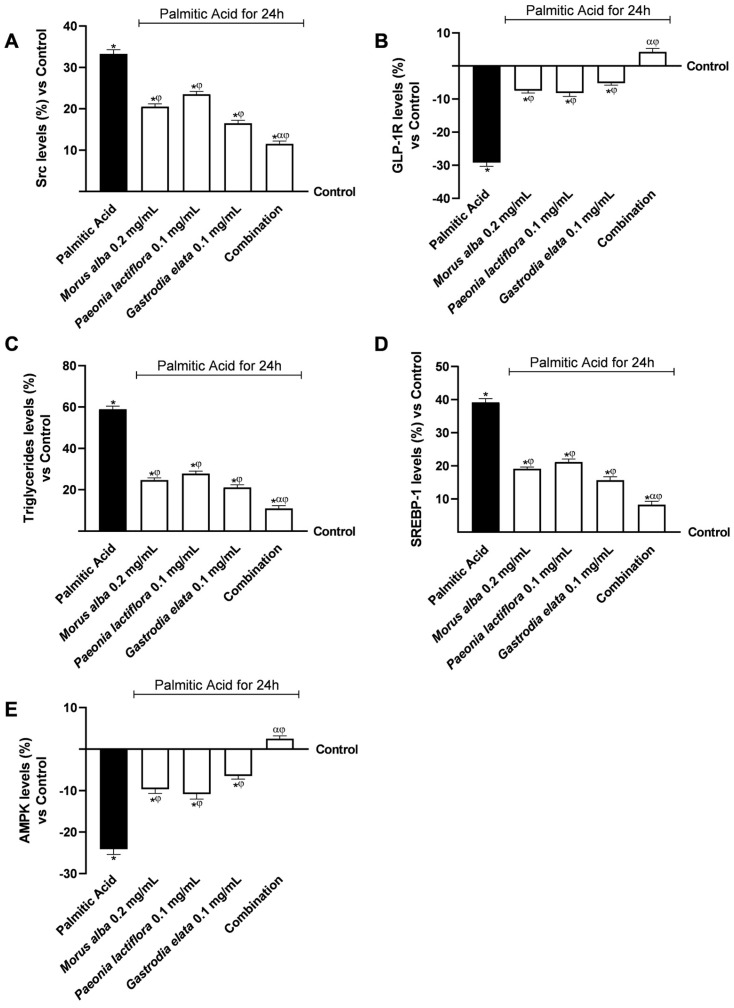
Protective effects against lipotoxicity markers in HepG2 cells. HepG2 cells were exposed to palmitic acid (PA) for 24 h to induce lipotoxicity followed by 24 h treatment with basolateral conditioned medium collected from the Transwell^®^ intestinal system. In (**A**–**E**) were reported the levels of signalling proteins and intracellular lipids quantified by ELISA: (**A**) Src, (**B**) GLP-1R, (**C**) triglycerides, (**D**) SREBP-1, and (**E**) AMPK. The *Morus alba* extract used in this study was obtained from leaves, the *Paeonia lactiflora* extract from roots, and the *Gastrodia elata* extract from rhizomes. Data are expressed as mean ± SD (%) of 5 independent experiments, each performed in triplicate, normalised to the control (0%) line. ^φ^ *p* < 0.05 vs. PA, * *p* < 0.05 vs. control, ^α^ *p* < 0.05 vs. single agents.

As shown in Figure 4, PA 0.5 mM exposure significantly altered hepatic metabolic signalling compared with control conditions (*p* < 0.05). Specifically, PA 0.5 mM markedly increased Src levels (+33.2%), a marker associated with alterations in metabolic and inflammatory signalling (Figure 4A). Conditioned media derived from intestinal cells treated with individual extracts partially attenuated Src upregulation, with *Gastrodia elata* 0.1 mg/mL exerting the strongest effect among the single agents. Notably, conditioned media from the combination produced a significantly greater reduction (*p* < 0.05), limiting Src levels to 11.4% above control levels, corresponding to an approximate 65% reduction relative to PA-treated cells.

PA 0.5 mM exposure also significantly decreased hepatic GLP-1R expression (−28.5%), indicating reduced responsiveness to incretin-mediated metabolic regulation (Figure 4B). While conditioned media from individual extracts partially restored GLP-1R expression (*p* < 0.05), media derived from the combination restored GLP-1R levels to values comparable to controls with a slight increase (+4.2%) under lipotoxic conditions. Appendix A (Table A1) shows that the actual combined effects exceeded the predicted additive responses under Bliss independence. Additionally, supplementary experiments including the GLP-1 receptor antagonist Exendin(9–39) 500 nM indicated partial attenuation of these effects, confirming a contributory role of GLP-1 receptor signalling in the observed downstream responses. In additional rescue experiments, it was observed that concomitant administration of exogenous GLP-1 or the GLP-1R agonist Liraglutide (100 nM) counteracted the effects of the antagonist, leading to a recovery of its levels (see Figure A1 and Figure A2 in the Appendix A).

Consistent with disrupted lipid handling, PA treatment induced a significant increase in intracellular TG accumulation (+58.7%; Figure 4C). Conditioned media from all extract-treated intestinal cells significantly reduced TG content (*p* < 0.05); however, the combination showed the greatest efficacy (α, *p* < 0.05), limiting TG accumulation to only 10.3% above control, compared with the 23.5% increase observed with the individual extracts.

To further assess hepatic lipogenic signalling, the expression of SREBP-1, a key transcriptional regulator of de novo lipogenesis, was evaluated (Figure 4D). Whereas conditioned media from single extracts modestly attenuated PA-induced SREBP-1 upregulation, the combination elicited a significantly stronger inhibitory effect, restricting SREBP-1 levels to 8.1% above control levels (*p* < 0.05) with a reduction of 73.6% compared to PA 0.5 mM (*p* < 0.05).

Finally, analysis of AMPK, a central energy sensor involved in metabolic homeostasis, revealed significant activation in response to all conditioned media treatments (Figure 4E). Conditioned media derived from the combination induced the most pronounced AMPK activation, exceeding baseline control levels by approximately 2.5%, consistent with enhanced hepatic energy sensing and reduced lipogenic signalling. The combination increased the AMPK levels by 1.20-fold compared to PA 0.5 mM (*p* < 0.05). Appendix A (Table A2) shows that the actual combined effects exceeded the predicted additive responses under Bliss independence. Raw data related to the results displayed in Figure 4B–D are provided in Appendix B (Table A5, Table A6 and Table A7). Additionally, supplementary experiments in Appendix A (Figure A2) using the GLP-1 receptor antagonist Exendin(9–39) 500 nM indicated partial attenuation of these effects for AMPK levels, confirming a contributory role of GLP-1 receptor signalling. Furthermore, in rescue experiments, it was observed that concomitant administration of exogenous GLP-1 or the GLP-1R agonist Liraglutide (100 nM) counteracted the effects of the antagonist, leading to a recovery of AMPK levels (see Figure A3 in the Appendix A).

**Figure 5:**
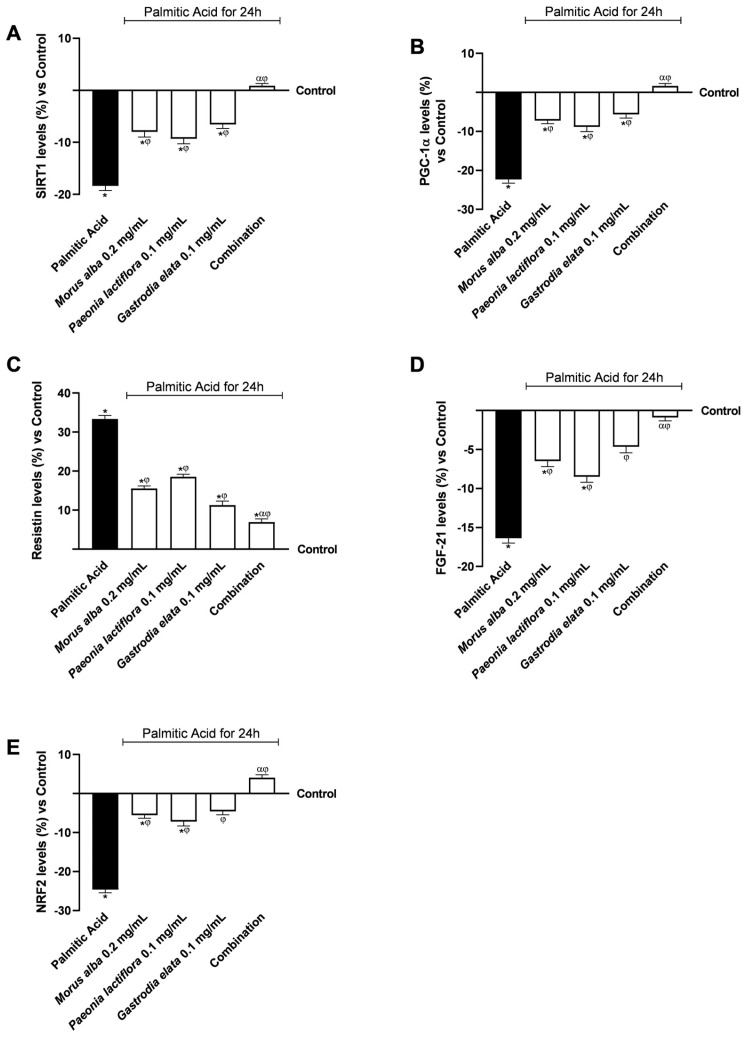
Comparative effects of individual extracts and their combination on metabolic signalling and oxidative stress markers. HepG2 cells were exposed to palmitic acid (PA) for 24 h to induce lipotoxicity followed by 24 h treatment with basolateral conditioned medium collected from the Transwell^®^ intestinal system. In (**A**–**E**) were reported the levels of signalling proteins quantified by ELISA assay: (**A**) SIRT1, (**B**) PGC1α, (**C**) Resistin, (**D**) FGF-21, and (**E**) NRF2. The *Morus alba* extract used in this study was obtained from leaves, the *Paeonia lactiflora* extract from roots, and the *Gastrodia elata* extract from rhizomes. Data are expressed as mean ± SD (%) from 5 independent experiments, each performed in triplicate and normalised to the control (0%) line. ^φ^ *p* < 0.05 vs. PA, * *p* < 0.05 vs. control, ^α^ *p* < 0.05 vs. single agents.

The impact of the intestinal conditioned media on hepatic metabolic flexibility was further assessed by quantifying key regulators of mitochondrial function, antioxidant defence, and inflammatory signalling in PA-challenged HepG2 cells. Additionally, the level of hepatokines was measured to evaluate the potential of the liver to modulate downstream systemic signals within this in vitro axis (Figure 5).

As shown in Figure 5A, PA exposure at 0.5 mM significantly reduced SIRT1 levels compared with control conditions (*p* < 0.05). Conditioned media derived from individual extracts partially attenuated this reduction but did not restore SIRT1 levels to baseline. In contrast, conditioned media from the combination was associated with a greater increase in SIRT1 levels (α, *p* < 0.05), normalising SIRT1 expression (+1.2% vs. control). The combination was also associated with a 1.12-fold increase in AMPK levels compared with PA 0.5 mM (*p* < 0.05).

A comparable trend was observed in PGC1α, a key regulator of mitochondrial oxidative metabolism (Figure 5B). While individual extract-derived conditioned media induced only partial recovery, the combined formulation fully restored PGC1α levels, reaching levels slightly above control (+1.4%) and increasing them by 1.10-fold compared to PA 0.5 mM.

PA 0.5 mM treatment significantly increased the expression of the pro-inflammatory hepatokine resistin (Figure 5C). All conditioned media treatments significantly reduced resistin levels relative to PA alone (*p* < 0.05); however, conditioned media from the combined formulation was associated with the largest reduction (α, *p* < 0.05), limiting the upregulation of resistin to 6.7%, compared to residual increases, averaging 17.05%, observed with individual extracts.

Conversely, PA exposure at 0.5 mM significantly increased FGF-21 levels (Figure 5D). Conditioned media derived from individual extracts partially mitigated this reduction, whereas media from the combination nearly normalised FGF-21 levels (−1.5% vs. control). The combination increased FGF-21 levels by 1.08-fold compared with PA at 0.5 mM (*p* < 0.05). Appendix A (Table A3) shows that actual combined effects exceeded predicted additive responses per Bliss independence.

Finally, NRF2, a key regulator of antioxidant responses, was significantly suppressed by PA 0.5 mM (Figure 5E). This effect was only modestly counteracted by individual extract-derived conditioned media. In contrast, conditioned media from the combined formulation was associated with an increase in NRF2 levels, slightly exceeding control levels (+4.8%, α, *p* < 0.05). The combination increased NRF2 levels by 1.15-fold compared with PA at 0.5 mM (*p* < 0.05).

Overall, intestinal-derived conditioned media from the combined plant extract formulation was associated with changes in multiple hepatic pathways related to mitochondrial function, inflammatory signalling, hepatokine expression, and antioxidant responses. Raw data related to the results displayed in Figure 5C,D are provided in Appendix B (Table A8 and Table A9). Additionally, supplementary experiments in Appendix A (Figure A2) using the GLP-1 receptor antagonist Exendin(9–39) at 500 nM indicated partial attenuation of the combined effects on resistin and SIRT1 levels, confirming a contributory role for GLP-1 receptor signalling. Furthermore, in rescue experiments, it was observed that concomitant administration of exogenous GLP-1 or the GLP-1R agonist Liraglutide (100 nM) counteracted the effects of the antagonist, leading to a recovery of resistin and SIRT1 levels (see Figure A3 in Appendix A).

### 3.5. Liver–Adipose Tissue Crosstalk: Botanical Formulations Promote Adipocyte Browning-Related Pathway and Mitigate Inflammation

**Figure 6:**
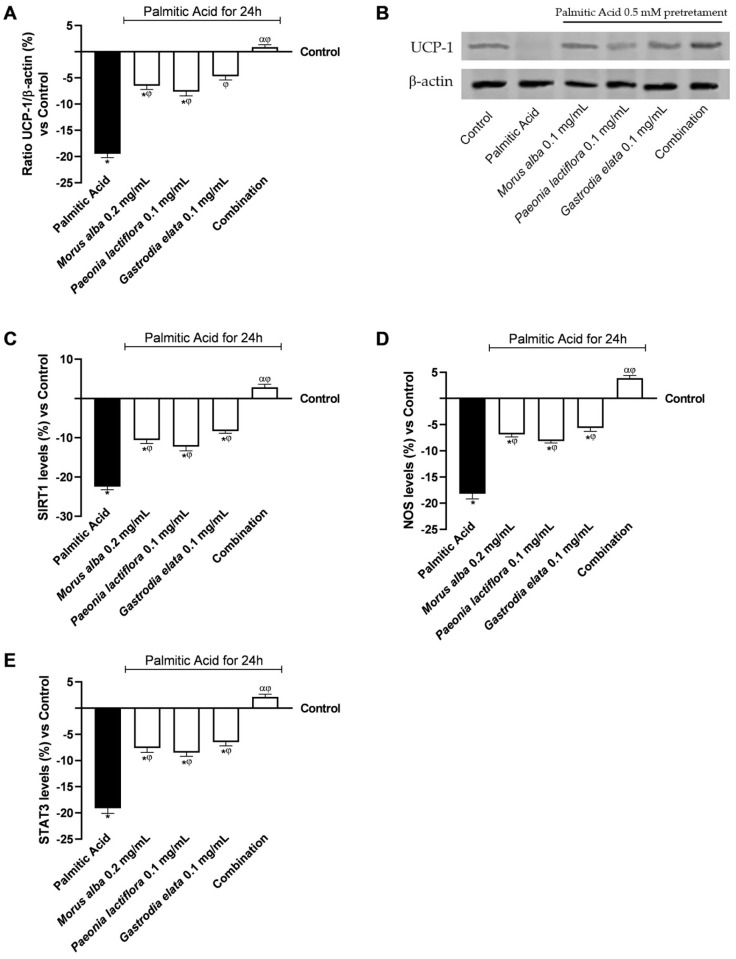
Effects of single extracts and their combination on browning-associated molecular pathways and inflammatory signalling. 3T3-L1 cells were exposed for 24 h with basolateral conditioned medium collected from the Transwell^®^ liver system. In (**A**,**B**) UCP1 analysis by Western blot: (**A**) densitometric quantification normalized to β-actin; (**B**) representative Western blot of UCP1. In (**C**–**E**) ELISA-based quantification of: (**C**) SIRT1, (**D**) NOS, and (**E**) STAT3 levels, respectively. The *Morus alba* extract used in this study was obtained from leaves, the *Paeonia lactiflora* extract from roots, and the *Gastrodia elata* extract from rhizomes. Data are expressed as mean ± SD (%) from 5 independent experiments, each performed in triplicate and normalised to the control (0%) line. For Western blot analysis, data are expressed as mean ± SD (%) from 3 independent experiments, each performed in triplicate and normalised to the control (0%) line. ^φ^ *p* < 0.05 vs. PA, * *p* < 0.05 vs. control, ^α^ *p* < 0.05 vs. single agents.

To assess whether liver-derived signals could potentially influence adipocyte metabolism, differentiated 3T3-L1 cells were exposed to media conditioned by BP-treated HepG2 cells (previously treated with intestinal basolateral supernatants). This setup allowed us to complete the in vitro reconstruction of the gut–liver–adipose axis and to study the botanical formulation’s ability to mitigate lipotoxicity-induced dysfunction within this multiorgan in vitro system (Figure 6).

As shown in Figure 6A,B, PA 0.5 mM exposure significantly decreased UCP1 expression compared with control adipocytes (*p* < 0.05), indicating impaired thermogenic capacity. Conditioned media derived from individual extract treatments partially restored UCP1 expression, whereas the combination was associated with levels close to or slightly above control (+1.8% vs. control). The combination increased UCP1 expression by 1.05-fold compared with PA at 0.5 mM (*p* < 0.05). The combination increased UCP1 expression by 1.05-fold compared with PA at 0.5 mM (*p* < 0.05). A post hoc analysis of Bliss independence was complemented by Appendix A (Table A4), which showed that the observed combined effects surpassed the expected additive responses. Furthermore, additional experiments in Appendix A (Figure A2) using the GLP-1 receptor antagonist Exendin(9–39) at 500 nM showed a partial reduction in the combination effects on UCP1 levels, measured via an ELISA kit, confirming the involvement of the GLP-1 receptor signalling.

SIRT1 levels were significantly reduced by PA 0.5 mM treatment (−18.5% vs. control; Figure 6C). Individual extract-conditioned media partially attenuated this reduction, while the combined formulation was associated with SIRT1 levels closer to control (−1.2%).

NOS levels, a marker of oxidative and inflammatory stress, were significantly elevated after PA exposure (+31.4% vs. control; Figure 6D). Conditioned media from the combination was associated with a smaller increase in NOS (3.5%), whereas individual treatments had a lesser effect. In detail, the combination increased NOS levels by 1.24-fold compared with PA at 0.5 mM (*p* < 0.05).

Phosphorylated STAT3, a key mediator of inflammatory signalling, was significantly increased in PA-treated adipocytes (+24.1% vs. control), thus limiting STAT3 levels (depicted in the dephosphorylated form in Figure 6E). All conditioned media treatments decreased STAT3 activation through phosphorylation (*p* < 0.05), with the combination associated with the greatest reduction, limiting STAT3 upregulation to 5.2% (α, *p* < 0.05). The combination increased STAT3 levels by 1.10-fold compared with PA at 0.5 mM (*p* < 0.05).

Collectively, hepatic-conditioned media derived from the combined botanical formulation was associated with changes in UCP1, SIRT1, NOS, and STAT3 levels in PA-challenged adipocytes, indicating improved thermogenic capacity, mitochondrial signalling, and lower inflammatory and oxidative stress markers at the cellular level. Furthermore, in rescue experiments, it was observed that concomitant administration of exogenous GLP-1 or the GLP-1R agonist Liraglutide (100 nM) counteracted the effects of the antagonist, leading to a recovery of UCP1 and SIRT1 levels (see Figure A3 in Appendix A). Raw data related to the results displayed in Figure 6A,C are provided in Appendix B (Table A10 and Table A11).

## 4. Discussion

Visceral fat accumulation plays a crucial role in metabolic dysregulation and results from the interaction of genetic, dietary, and environmental factors that disrupt energy homeostasis and inter-organ communication [73]. Excess lipid storage is closely associated with low-grade inflammation, impaired lipid processing, and dysfunction of the gut–liver–adipose axis, an essential regulatory network that controls metabolic flexibility and energy expenditure [73,74,75,76]. Within this axis, the central role of the intestinal barrier in regulating nutrient absorption and enteroendocrine signalling is highlighted, thereby influencing downstream hepatic and adipose metabolic responses [77,78]. Among gut-derived hormones, GLP-1 is a key mediator linking nutrient sensing to systemic metabolic regulation. Reduced endogenous GLP-1 secretion and impaired GLP-1R responsiveness have consistently been associated with obesity, insulin resistance, and increased lipid storage, whereas restoring GLP-1 signalling improves metabolic flexibility and adiposity [79].

In this study, the combination of plant-derived extracts induced coordinated metabolic effects along the gut–liver–adipose axis, with changes consistent with involvement of GLP-1-associated signalling. At the intestinal level, the combined formulation maintains epithelial integrity in Caco-2 cells, as indicated by stable TEER values and increased TJ protein expression, and promotes GLP-1 secretion. These cellular-level observations are consistent with previous reports that dietary bioactives can stimulate endogenous GLP-1 release in vitro [80] and suggest that the botanical mixture may produce complementary effects compared to individual extracts.

Importantly, intestinal-derived signals produced by the combined treatment were associated with significant hepatic metabolic adaptations under lipotoxic conditions. In HepG2 cells, exposure to conditioned media from the intestinal model resulted in decreased intracellular TG accumulation and reduced lipogenic signalling, as evidenced by downregulation of SREBP-1. These effects were accompanied by the restoration of GLP-1R expression and a decrease in Src activation through phosphorylation, a kinase involved in insulin resistance and metabolic inflammation [81]. Overall, these in vitro findings suggest that increased intestinal GLP-1 availability may modulate hepatic signalling pathways, including lipid metabolism, at the cellular level, without implying direct in vivo metabolic effects.

Concurrent activation of AMPK and the restoration of SIRT1 and PGC1α further support the development of a metabolically beneficial hepatic phenotype, characterised by improved mitochondrial function and oxidative metabolism [82,83]. The regulation of hepatokines supports this finding: resistin, linked to inflammation and lipid accumulation, was decreased, while FGF-21, a vital regulator of fatty acid oxidation and energy expenditure, was restored to near-normal levels [84,85]. These coordinated changes position the liver as a key relay through which intestinal signals modify systemic metabolic responses.

Downstream effects of these hepatic adaptations were evident in adipocytes exposed to hepatic-conditioned media. In differentiated 3T3-L1 cells, the botanical combination modulated inflammatory and oxidative stress markers, including STAT3 and NOS, and influenced browning-related signalling pathways, such as UCP1, without direct measurement of thermogenic activity or energy expenditure. The browning of white adipose tissue, characterised by enhanced mitochondrial uncoupling and energy dissipation, is increasingly recognised as a key mechanism for reducing lipid storage and improving metabolic health [86,87]. The observed upregulation of UCP1 and related markers suggests that hepatic-derived signals produced following combined botanical treatment modulate browning-related signalling pathways in adipocytes, without implying functional thermogenesis. The concurrent activation of the AMPK/SIRT1/PGC1α axis in both hepatic and adipose models further supports the involvement of mitochondrial biogenesis and oxidative metabolism as common downstream effectors of the observed metabolic enhancements [14].

Similar browning-related signalling effects have been reported for the botanical extracts tested. *Paeonia lactiflora* root increased UCP1 and browning-associated markers, such as PGC1α, in 3T3-L1 adipocytes via AMPK activation, supporting enhanced mitochondrial uncoupling and energy expenditure [88]. Moreover, combined extracts of *Morus alba* leaf and root improved browning-related signalling activation in vitro, including UCP1, and activated the AMPK-SIRT1-UCP1 axis in adipocyte models, consistent with greater-than-additive metabolic effects [89]. Natural phenolic compounds have also been shown to increase and regulate the expression of UCP1 and other thermogenic markers in 3T3-L1 cells, indicating a broader potential for plant-derived agents to promote adipocyte browning [90]. These observations underscore the utility of the 3T3-L1 cell model for studying adipocyte thermogenic programming. Indeed, 3T3-L1 adipocytes faithfully recapitulate key molecular pathways involved in mitochondrial uncoupling, browning, and sirtuin-mediated energy regulation [91]. This makes them particularly suitable for investigating the effects of hepatic-derived signals and botanical treatments, such as *Paeonia lactiflora* and *Morus alba*, on adipocyte thermogenic activation and inflammatory response.

Notably, the botanical formulation showed combined effects greater than those of individual extracts, which were consistently observed across some experimental levels, indicating that functional complementarity among the botanical components is necessary to achieve coordinated metabolic adaptations across the gut–liver–adipose axis. The enhanced effects of the combined formulation can be explained by the complementary phytochemical profiles and multi-target actions of the three botanical extracts. *Morus alba*, rich in 1-DNJ and polysaccharides, contributes to the modulation of carbohydrate metabolism and glycaemic control [92], *Gastrodia elata* provides polysaccharides and polyphenols with anti-inflammatory and metabolic regulatory properties [93], while *Paeonia lactiflora* offers polyphenols and flavonoids associated with AMPK activation to lipid metabolism and GLP-1 signalling [88]. The combination may support coordinated modulation along the gut–liver–adipose axis, potentially enhancing interactions among different compartments and producing broader effects than those observed with individual extracts. The experimental responses likely reflect the integrated activity of the multi-component formulation of the multi-component formulation, in which distinct bioactive molecules appear to act in a complementary manner across the different experimental models. Notably, the use of the GLP-1R antagonist Exendin(9–39) partially or significantly abrogates these effects, supporting the hypothesis of an integrated regulatory mechanism in which intestinal signalling influences adipocyte browning pathways through a receptor-dependent process. This inhibition was further characterised by a salvage approach; administration of exogenous GLP-1 or the GLP-1R agonist Liraglutide was able in HepG2 and 3T3-L1 (with PA pretreatment) to restore AMPK, SIRT1, resistin levels and at the same time UCP-1 and SIRT1 levels in adipocytes, which had been reduced by the antagonist. These observations underscore the role of the GLP-1R-mediated pathway in bridging intestinal signalling with hepatic and adipocyte metabolic responses within this in vitro model. In this perspective, GLP-1R can act as a central molecular hub through which the overlapping yet complementary activities of the extracts may contribute to integrated regulation of metabolic processes, from intestinal barrier function and GLP-1 secretion to browning-related signalling in adipocytes. While direct assessment of body weight or adipose mass is not feasible in vitro, the observed changes reflect early molecular and signalling events at the cellular level. They provide mechanistic insight but cannot be extrapolated to predict systemic or clinical outcomes [94]. Similar effects have been reported for nutraceutical interventions that act through GLP-1- and AMPK-dependent mechanisms, including *Morus alba*-derived bioactives [95], supporting the translational relevance of the present findings.

Several limitations should be acknowledged. This study relies entirely on in vitro models, which cannot fully replicate systemic endocrine, neural, or immune interactions. Although Caco-2, HepG2, and 3T3-L1 cells are well-established models for investigating intestinal, hepatic, and adipose functions [96,97,98], they provide mechanistic insight at the cellular level rather than direct evidence of physiological or clinical effects. Nonetheless, the integrated multi-organ approach employed here allowed a detailed examination of early molecular events linking intestinal function with hepatic metabolism and browning-related signalling in adipocytes, generating testable hypotheses for future in vivo validation.

In conclusion, this study offers mechanistic evidence that a combination of plant-derived extracts can influence GLP-1-associated signalling and coordinate metabolic adaptations along the gut–liver–adipose axis. The observed greater-than-additive effects among the botanical components highlight the potential of multi-target nutraceutical strategies to modulate metabolic flexibility and pathways involved in lipid handling and adipocyte remodelling. However, these findings primarily reflect mechanistic intercellular signalling observed under controlled in vitro conditions and do not provide direct evidence of effects on body weight, visceral fat, energy expenditure, or functional thermogenesis in vivo. It is important to note that factors such as intestinal absorption, first-pass metabolism, systemic distribution, and achievable plasma concentrations of the bioactive compounds are not accounted for in the current models. Therefore, while modulation of endogenous GLP-1 signalling by these plant-derived extracts remains biologically plausible, the translational relevance of these observations should be interpreted with caution.

Future studies employing in vivo models and clinical investigations will be essential to validate these mechanisms, determine physiologically relevant doses, and assess their potential impact on systemic metabolic outcomes.

## 5. Conclusions

This study provides mechanistic insight into GLP-1-associated signalling and related cellular responses within an integrated in vitro gut–liver–adipose model. Within this experimental framework, a standardised combination of extracts from *Gastrodia elata*, *Morus alba*, and *Paeonia lactiflora* increased intestinal GLP-1 secretion and induced downstream changes in cellular pathways related to hepatic lipid metabolism, hepatokine signalling, and adipocyte function.

Across sequential models, the combined formulation consistently showed greater effects than individual extracts, suggesting greater-than-additive effects across the experimental models, although formal pharmacological synergy analyses were not performed. At the hepatic level, intestinal-derived conditioned media were associated with modulation of pathways involved in metabolic flexibility, including the AMPK/SIRT1/PGC1α axis, as well as changes in intracellular triglyceride levels and hepatokine-related markers. These effects were paralleled in adipocytes by reduced lipid accumulation and modulation of markers linked to inflammatory signalling and cellular remodelling.

Given the in vitro nature of this study, these findings should be interpreted as reflecting cellular and molecular responses rather than whole-body physiological regulation and are therefore mechanistic and hypothesis-generating. Overall, this work supports the utility of integrated multi-organ in vitro systems to study intercellular metabolic communication and nutrient-responsive signalling at the mechanistic level.

## Figures and Tables

**Table 1:** Content of the main bioactive components in the plant-derived extracts. Values are expressed as mg/g of dry extract and reported as mean ± SD of five independent experiments, each performed in triplicate.

Sample	Methods	Components	Content (%)
*Morus alba* (Leaf extract)	HPLC	1-DNJ	5.03 ± 0.26
	Phenol–sulfuric acid	Total polysaccharides	12.00 ± 0.94
*Paeonia lactiflora* (Root extract)	Folin–Ciocalteu	Total polyphenols	5.00 ± 0.43
	AlCl_3_	Total flavonoids	0.70 ± 0.17
*Gastrodia elata* (Rhizome extract)	Phenol–sulfuric acid	Total polysaccharides	10.20 ± 0.82
	Folin–Ciocalteu	Total polyphenols	2.00 ± 0.34

## Data Availability

Data are available from the corresponding author upon reasonable request and for justified scientific reasons, since they relate to a patented substance.

## Abbreviations

The following abbreviations are used in this manuscript:

1-DNJ	1-deoxynojirimycin
Adv-DMEM	Advanced Dulbecco’s Modified Eagle Medium
AMPK	AMP-activated protein kinase
ATCC	American Type Culture Collection
DMEM	Dulbecco’s Modified Eagle’s Medium
ELISA	Enzyme-Linked Immunosorbent Assay
EMA	European Medicines Agency
eNOS	Endothelial nitric oxide synthase
FBS	Fetal bovine serum
FDA	Food and Drug Administration
FGF21	Fibroblast growth factor 21
GLP-1	Glucagon-like peptide-1
GLP-1R	GLP-1 receptor
HPLC	High-performance liquid chromatography
HRP	Streptavidin-horseradish peroxidase
MTT	3-(4,5-dimethylthiazol-2-yl)-2,5-diphenyltetrazolium bromide
NRF2	Nuclear factor erythroid 2–related factor 2
PA	Palmitic Acid
PGC1α	Peroxisome proliferator-activated receptor γ coactivator 1α
PVDF	Polyvinylidene difluoride
RPMI	Roswell Park Memorial Institute
SD	Standard Deviation
SDS-PAGE	Sodium Dodecyl Sulphate-PolyAcrylamide Gel Electrophoresis
SIRT1	Sirtuin 1
Src	Proto-oncogene tyrosine-protein kinase Src
SREBP-1c	Sterol regulatory element-binding protein 1c
STAT3	Signal transducer and activator of transcription 3
TEER	Transepithelial electrical resistance
TG	Triglyceride
TJ	Tight junction
TMB	3,3′,5,5′-tetramethylbenzidine
UCP1	Uncoupling protein 1
ZO-1	Zonula Occludens-1

## Appendix A

### Appendix A.1. Determination of 1-DNJ by HPLC

The quantification of 1-DNJ in *Morus alba* extract was performed by HPLC (Agilent, Santa Clara, CA, USA), a method known for its sensitivity and reproducibility in the determination of iminosugars in botanical matrices. Dried *Morus alba* samples were finely ground and extracted with ultrapure water or mildly acidic buffer (pH 4–5) under constant agitation at room temperature. Extracts were subsequently centrifuged and filtered through 0.22 μm membrane filters before analysis.

To enhance detectability, 1-DNJ was derivatised with 9-fluorenylmethyl chloroformate (FMOC-Cl) before chromatographic separation. Separation was achieved on a C18 reversed-phase column (250 mm × 4.6 mm, 5 μm) using a gradient mobile phase consisting of acetonitrile and water containing 0.1% trifluoroacetic acid (TFA), at a flow rate of 1 mL/min and a column temperature of 30 °C. Detection was carried out at 254 nm. Quantification was based on calibration curves generated using derivatised DNJ standards, and results were expressed as mg 1-DNJ per g of dried plant material.

The method was validated for linearity (R^2^ ≥ 0.999), precision, and accuracy. Limits of detection (LOD = 3) and quantification (LOQ = 10) were calculated based on signal-to-noise ratios. Derivatised samples were stored in the dark at 4 °C and analysed within 24 h. This analytical protocol is consistent with previously reported methods for the determination of DNJ in *Morus alba* extracts.

### Appendix A.2. Determination of Total Polysaccharide Content

The total polysaccharide content of *Gastrodia elata* extract was determined using the phenol–sulfuric acid colourimetric method, as previously described [40]. Briefly, aliquots of the extract were dissolved in purified water and diluted to fall within the assay’s linear range. One millilitre of sample solution was mixed with 1.0 mL of 5% (*w*/*v*) phenol solution, followed by the rapid addition of 5.0 mL of concentrated sulfuric acid.

The reaction mixture was incubated at room temperature for 30 min to allow colour development, and absorbance was measured at 490 nm using a UV–Vis spectrophotometer. D-glucose was used as the reference standard for calibration, and total polysaccharide content was expressed as mean (%) ± SD. All measurements were performed in triplicate.

### Appendix A.3. Determination of Total Flavonoid Content

Total flavonoid content (TFC) in *Paeonia lactiflora* extract was determined using a previously established aluminium chloride-based colourimetric method [42]. Briefly, 1.0 mL of extract solution (0.1 mg/mL) was mixed with 4.0 mL of distilled water in a 10 mL volumetric flask. Subsequently, 0.3 mL of 5% NaNO_2_ solution was added, followed by 0.3 mL of 10% AlCl_3_ after 5 min. After an additional 6 min, 2.0 mL of 1 M NaOH was added, and the volume was adjusted to 10 mL with distilled water (all reagents from Merck Life Science, Rome, Italy).

After thorough mixing, absorbance was measured at 510 nm. Results were expressed as mean (%) ± SD of triplicate measurements.

### Appendix A.4. Determination of Total Polyphenol Content

Total polyphenol content (TPC) was measured using the Folin–Ciocalteu colourimetric method, following established protocols with minor modifications [43]. Aliquots of *Gastrodia elata* and *Paeonia lactiflora* extracts were mixed with the Folin–Ciocalteu reagent (Merck Life Science, Rome, Italy) and incubated at room temperature for 3–5 min. Afterwards, an aqueous sodium carbonate solution was added to alkalinise the reaction mixture. Samples were kept in the dark at room temperature for 30–60 min to develop colour. Absorbance was read at 760 nm using a UV–Vis spectrophotometer. Gallic acid served as the reference standard, and the results were expressed as mean ± SD from at least three independent experiments.

### Appendix A.5. Cell Viability Assay

Cell viability was evaluated using an MTT-based In Vitro Toxicology Assay Kit (Merck Life Science, Rome, Italy) on 96-well plates, following standard procedures previously described in the literature [55]. Briefly, cells were seeded at suitable densities and exposed to the tested extracts and their combination for specified time periods. At the end of the treatment, MTT reagent was added and incubated to enable formazan crystal formation. The formed formazan was solubilised, and absorbance was measured at 570 nm with background correction at 690 nm using a microplate reader (Infinite 200 Pro MPlex, Tecan, Männedorf, Switzerland). Cell viability was expressed as a percentage relative to untreated control cells, set as 100%. Data are presented as the mean ± SD of five independent experiments conducted in triplicate.

**Table A1:** Bliss independence analysis on GLP-1R levels.

Treatment	Expected Data	Observed Data	Interaction
Combination	−22.40%	4.30%	Synergistic

**Table A2:** Bliss independence analysis on AMPK levels.

Treatment	Expected Data	Observed Data	Interaction
Combination	−29.60%	2.50%	Synergistic

**Table A3:** Bliss independence analysis on FGF-21 levels.

Treatment	Expected Data	Observed Data	Interaction
Combination	−20.97%	−0.90%	Synergistic

**Table A4:** Bliss independence analysis on UCP-1 expression.

Treatment	Expected Data	Observed Data	Interaction
Combination	−20.03%	0.78%	Synergistic

## Appendix B

**Table A5:** Effects of different treatments on GLP-1R levels in vitro. Raw data (ng/mL) corresponding to the graph shown in Figure 4B. Values are expressed as mean ± SD of five independent experiments performed in triplicate. Percentage change indicates the difference relative to the Control.

Samples	GLP1-R (ng/mL) Mean ± SD	% Difference vs. Control
Control	7.80 ± 0.25	
Palmitic Acid	5.52 ± 0.30	−29.20
*Morus alba* 0.2 mg/mL	7.21 ± 0.28	−7.50
*Paeonia lactiflora* 0.1 mg/mL	7.16 ± 0.27	−8.20
*Gastrodia elata* 0.1 mg/mL	7.39 ± 0.26	−5.20
Combination	8.14 ± 0.29	4.30

**Table A6:** Effects of different treatments on TG levels in vitro. Raw data (mmol/L) corresponding to the graph shown in Figure 4C. Values are expressed as mean ± SD of five independent experiments performed in triplicate. Percentage change indicates the difference relative to the Control.

Samples	TG (mmol/L) Mean ± SD	% Difference vs. Control
Control	5.34 ± 0.22	
Palmitic Acid	8.49 ± 0.35	58.90
*Morus alba* 0.2 mg/mL	6.66 ± 0.25	24.70
*Paeonia lactiflora* 0.1 mg/mL	6.83 ± 0.29	27.85
*Gastrodia elata* 0.1 mg/mL	6.47 ± 0.27	21.10
Combination	5.92 ± 0.25	10.95

**Table A7:** Effects of different treatments on SREBP-1 levels in vitro. Raw data (ng/mL) corresponding to the graph shown in Figure 4D. Values are expressed as mean ± SD of five independent experiments performed in triplicate. Percentage change indicates the difference relative to the Control.

Samples	SREBP-1 (ng/mL) Mean ± SD	% Difference vs. Control
Control	12.00 ± 0.50	
Palmitic Acid	16.70 ± 0.69	39.20
*Morus alba* 0.2 mg/mL	14.29 ± 0.25	19.10
*Paeonia lactiflora* 0.1 mg/mL	14.54 ± 0.62	21.15
*Gastrodia elata* 0.1 mg/mL	13.88 ± 0.58	15.69
Combination	12.99 ± 0.55	8.25

**Table A8:** Effects of different treatments on resistin levels in vitro. Raw data (pg/mL) corresponding to the graph shown in Figure 5C. Values are expressed as mean ± SD of five independent experiments performed in triplicate. Percentage change indicates the difference relative to the Control.

Samples	Resistin (pg/mL) Mean ± SD	% Difference vs. Control
Control	764.0 ± 5.81	
Palmitic Acid	1020.3 ± 6.22	33.35
*Morus alba* 0.2 mg/mL	882.1 ± 5.69	15.53
*Paeonia lactiflora* 0.1 mg/mL	905.3 ± 6.12	18.48
*Gastrodia elata* 0.1 mg/mL	850.2 ± 5.61	11.26
Combination	815.2 ± 5.92	6.88

**Table A9:** Effects of different treatments on FGF-21 levels in vitro. Raw data (ng/mL) corresponding to the graph shown in Figure 5D. Values are expressed as mean ± SD of five independent experiments performed in triplicate. Percentage change indicates the difference relative to the Control.

Samples	FGF-21 (ng/mL) Mean ± SD	% Difference vs. Control
Control	838.0 ± 5.42	
Palmitic Acid	701.0 ± 5.80	−16.36
*Morus alba* 0.2 mg/mL	783.2 ± 6.13	−6.54
*Paeonia lactiflora* 0.1 mg/mL	767.1 ± 5.91	−8.50
*Gastrodia elata* 0.1 mg/mL	798.2 ± 5.53	−4.65
Combination	830.5 ± 5.74	−0.90

**Table A10:** Effects of different treatments on UCP-1 expression in vitro. Raw data of ratio UCP-1/β-actin corresponding to the graph shown in Figure 6A. Values are expressed as mean ± SD of five independent experiments performed in triplicate. Percentage change indicates the difference relative to the Control.

Samples	UCP-1/β-Actin Mean ± SD	% Difference vs. Control
Control	0.960 ± 0.05	
Palmitic Acid	0.773 ± 0.06	−19.47
*Morus alba* 0.2 mg/mL	0.897 ± 0.05	−6.45
*Paeonia lactiflora* 0.1 mg/mL	0.886 ± 0.07	−7.72
*Gastrodia elata* 0.1 mg/mL	0.915 ± 0.08	−4.68
Combination	0.968 ± 0.03	0.78

**Table A11:** Effects of different treatments on SIRT1 levels in vitro. Raw data (ng/mL) corresponding to the graph shown in Figure 6C. Values are expressed as mean ± SD of five independent experiments performed in triplicate. Percentage change indicates the difference relative to the Control.

Samples	SIRT1 (ng/mL) Mean ± SD	% Difference vs. Control
Control	132.0 ± 6.21	
Palmitic Acid	102.5 ± 5.50	−22.45
*Morus alba* 0.2 mg/mL	117.9 ± 5.08	−10.62
*Paeonia lactiflora* 0.1 mg/mL	115.9 ± 5.43	−12.26
*Gastrodia elata* 0.1 mg/mL	121.1 ± 5.28	−8.30
Combination	135.8 ± 5.62	2.85
